# Identification of a molecular locus for normalizing dysregulated GABA release from interneurons in the Fragile X brain

**DOI:** 10.1038/s41380-018-0240-0

**Published:** 2018-09-17

**Authors:** Yi-Mei Yang, Jason Arsenault, Alaji Bah, Mickael Krzeminski, Adam Fekete, Owen Y. Chao, Laura K. Pacey, Alex Wang, Julie Forman-Kay, David R. Hampson, Lu-Yang Wang

**Affiliations:** 1 Department of Biomedical Sciences, University of Minnesota Medical School, 1035 University Drive, Duluth, MN 55812 USA; 2 Program in Neurosciences & Mental Health, SickKids Research Institute, Toronto, ON M5G 1X8 Canada; 3 Department of Physiology, Faculty of Medicine, University of Toronto, Toronto, Canada; 4 Department of Pharmaceutical Sciences, Leslie Dan Faculty of Pharmacy, University of Toronto, Toronto, Canada; 5 Program in Molecular Medicine, SickKids Research Institute, Toronto, ON M5G 1X8 Canada; 6 Department of Biochemistry, Faculty of Medicine, University of Toronto, Toronto, Canada; 7 Department of Pharmacology, Faculty of Medicine, University of Toronto, Toronto, Canada

**Keywords:** Neuroscience, Physiology

## Abstract

Principal neurons encode information by varying their firing rate and patterns precisely fine-tuned through GABAergic interneurons. Dysregulation of inhibition can lead to neuropsychiatric disorders, yet little is known about the molecular basis underlying inhibitory control. Here, we find that excessive GABA release from basket cells (BCs) attenuates the firing frequency of Purkinje neurons (PNs) in the cerebellum of Fragile X Mental Retardation 1 (*Fmr1*) knockout (KO) mice, a model of Fragile X Syndrome (FXS) with abrogated expression of the Fragile X Mental Retardation Protein (FMRP). This over-inhibition originates from increased excitability and Ca^2+^ transients in the presynaptic terminals, where Kv1.2 potassium channels are downregulated. By paired patch-clamp recordings, we further demonstrate that acutely introducing an N-terminal fragment of FMRP into BCs normalizes GABA release in the *Fmr1*-KO synapses. Conversely, direct injection of an inhibitory FMRP antibody into BCs, or membrane depolarization of BCs, enhances GABA release in the wild type synapses, leading to abnormal inhibitory transmission comparable to the *Fmr1*-KO neurons. We discover that the N-terminus of FMRP directly binds to a phosphorylated serine motif on the C-terminus of Kv1.2; and that loss of this interaction in BCs exaggerates GABA release, compromising the firing activity of PNs and thus the output from the cerebellar circuitry. An allosteric Kv1.2 agonist, docosahexaenoic acid, rectifies the dysregulated inhibition in vitro as well as acoustic startle reflex and social interaction in vivo of the *Fmr1*-KO mice. Our results unravel a novel molecular locus for targeted intervention of FXS and perhaps autism.

## Introduction

Neurons communicate through intricate synaptic connections. Although excitatory inputs drive postsynaptic firing, the firing rate and patterns are strongly regulated by inhibition from GABAergic interneurons in the network. Depending on the spatiotemporal balance between excitation and inhibition (E/I balance), principal neurons optimize their coding capacity by varying spike frequencies. E/I balance is critical for circuit development, synaptic plasticity, learning, and memory. Conversely, E/I imbalance causes neurological disorders including autism, schizophrenia, and epilepsy [1–3]. Despite tremendous progress in understanding the elements underlying excitation, much less is known about the molecular mechanisms engaged in the dynamic control of inhibition.

Inhibition in the olivocerebellar system exemplifies the importance of interneurons in coordinating motor learning and cognitive processing [4, 5]. Being the sole output of the cerebellar cortex, Purkinje neurons (PNs) integrate multisensory excitatory inputs from climbing fibers (CFs) and parallel fibers (PFs), while GABAergic interneurons in the molecular layer, primarily basket cells (BCs) and stellate cells (SCs), exert powerful inhibition at the soma and distal dendrites of PNs, respectively, to fine-tune their firing. Through extensive cerebello-thalamo-cortical loops, aberrant activity in PNs can affect the development and function of brain networks and lead to neurodevelopmental disorders, such as autism [6–9]. In support of this view, Tsai et al. [10] have shown that PN-specific deletion of tuberous sclerosis complex 1 (Tsc1) reduces the excitability and spontaneous firing of PNs, resulting in system-wide autistic-like social and non-social behaviors. These are reminiscent of phenotypic traits of Fragile X Mental Retardation 1 (*Fmr1*) knockout (KO) mice, a model for Fragile X Syndrome (FXS), which is associated with a loss of an mRNA binding protein, Fragile X Mental Retardation Protein (FMRP) [11–13].

Although insightful studies highlight the importance of the cerebral cortex and hippocampus in neuropathology of FXS where a lack of FMRP impairs synaptic plasticity and development [14–16], restoring FMRP in the forebrain cannot fully rescue the FXS phenotypes [17, 18]. In other brain regions, particularly the cerebellum, reduced PN number and/or activity appear to be common between FXS and autism [10, 19, 20]. We examine the cerebellar circuitry in the *Fmr1*-KO mice and demonstrate that deletion of *Fmr1* significantly lowers the spontaneous firing rate of PNs because of increased GABA release from interneuron. We further delineate that the N-terminus of FMRP directly binds to the phosphorylated serine motif in the C-terminus of Kv1.2, a low threshold potassium channel that is particularly abundant in fast-spiking interneurons. This interaction critically regulates the activity of Kv1.2 and excitability of BC nerve terminals, controlling GABA release and thereby the firing frequency of PNs. Importantly, we find docosahexaenoic acid (DHA), a major component of omega-3 fatty acids, which serves as an allosteric agonist for Kv1.2, rectifies the synaptic deficits, elevated startle response, and impaired social interaction in the *Fmr1*-KO mice.

## Methods summary

Details are provided in the Supplementary Materials and Methods.

## Results

### Excessive GABA release from interneurons suppresses firing of PNs in the *Fmr1*-KO cerebellum

**Fig. 1:**
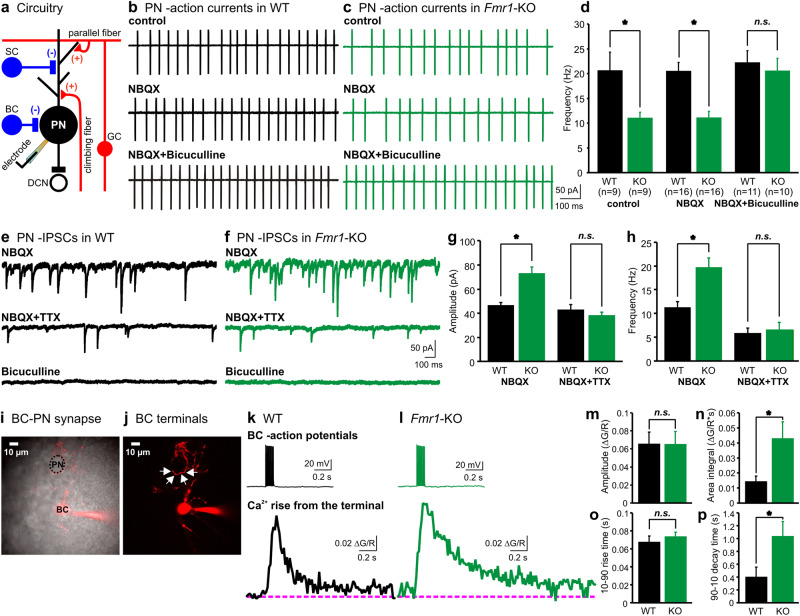
Reduced activity of PNs by elevated Ca^2+^ influx and GABA release from interneuron nerve terminals in *Fmr1*-KO synapses. **a** Schematics of the cerebellar circuitry including excitatory and inhibitory inputs to a PN. **b**, **c** Representative firing activity of PNs recorded in the cell-attached mode from WT (**b**) and *Fmr1*-KO (**c**) cerebellar slices in the absence (control, top panels) or presence of an AMPA receptor blocker NBQX (10 μM, middle panels), or in a combination of NBQX and a GABA_A_ receptor blocker bicuculline (10 μM, bottom panels). **d** Summary of the frequency of APs from WT (black bars) and KO (green bars) PNs in the aforementioned conditions (**b**, **c**). **e**, **f** IPSCs obtained in the whole-cell mode at a holding potential of −60 mV from PNs of WT (**e**) and KO (**f**) mice in NBQX to block excitatory inputs. The same PNs are subsequently exposed to a Na^+^ channel blocker TTX (1 μM) and bicuculline (10 μM). **g**, **h** Summary of the amplitude and frequency of IPSCs recorded from the WT (black bars, *n* = 23) and KO (green bars, *n* = 18) animals in the above conditions (**e**, **f**). **i**, **j** Images of a BC filled with Alexa Fluor 594 (30 µM, red) projecting a long axon to innervate a PN (grey). Arrows indicate the boutons of the BC nerve terminal in a 3D multiphoton image. **k**, **l** Averaged intra-terminal Ca^2+^ signals (bottom panels) from 5 trials evoked by AP trains (100 Hz, 100 ms, top panels) delivered to the soma of BCs in WT (**k**) and KO (**l**) slices. **m**–**p** Summary of the amplitude (**m**), area integral (**n**), 10–90 rise time (**o**), and 90–10 decay time (**p**) of Ca^2+^ transients measured from the WT (black bars, *n* = 6) and KO (green bars, *n* = 7) nerve terminals. In this and following figures, data are represented as mean ± s.e.m. and asterisks (*) denote statistical significance (*p* < 0.05)

Previous work in the *Fmr1*-KO mice shows that long-term depression (LTD) is enhanced compared to wild-type (WT) mice at several excitatory synapses including the PF–PN synapse in the cerebellum [21–23]. However, it is unclear whether and how FMRP regulates inhibition from presynaptic interneurons. We addressed this question in the cerebellar circuitry where SCs and BCs extend inhibitory projections onto PNs to counteract excitatory inputs from PFs and CFs (Fig. 1a). To examine the influences of synaptic inputs on the firing of PNs, we made cell-attached patch clamp recordings of spontaneous spikes from PNs in the vermal folia V & VI of sagittal cerebellar slices. Fig. 1b–d revealed a marked reduction in the firing rate of *Fmr1*-KO as compared to WT neurons. On average, the spike frequency decreased by 50%. Addition of NBQX, a blocker of AMPA receptors, had little effect on the spontaneous firing of either WT or KO PNs. When a GABA_A_ receptor antagonist bicuculline was co-applied, the differences in PN firing rates were abolished; indicating that the intrinsic excitability of PNs remained unaltered. The attenuated spontaneous firing of *Fmr1*-KO PNs is likely mediated by GABAergic inhibition from interneurons.

When we isolated spontaneous excitatory postsynaptic currents (sEPSCs) in bicuculline by whole-cell voltage clamping PNs, we observed no difference in their amplitude or frequency between WT and KO neurons (Supplementary Figure 1a-b). In contrast, we found spontaneous inhibitory postsynaptic currents (sIPSCs) recorded in NBQX from the *Fmr1*-KO slices were much more frequent and larger than those from the WTs (Fig. 1e–h). Upon inhibiting presynaptic action potentials (APs) with tetrodotoxin (TTX), a Na^+^ channel blocker, to obtain miniature inhibitory postsynaptic currents (mIPSCs), we noted that the amplitude and frequency of mIPSCs became indistinguishable between the two groups, implying that presynaptic quantal release and postsynaptic GABA_A_ receptors were not affected by knockout of *Fmr1*. Application of bicuculline eliminated all the events, confirming that they originated from GABAergic inputs. Therefore, we conclude that the enhanced IPSCs in the *Fmr1*-KO synapses are driven by AP-evoked GABA release from presynaptic interneurons.

Among the interneurons, BCs are known to provide predominant GABAergic inhibition to clamp spontaneous activity of PNs from large terminals with numerous release sites onto the soma, and by controlling spike initiation via ephaptic connection to the axon initial segment of PNs [24]. Therefore, one possible reason for the reduced activity of PNs in the *Fmr1*-KO synapses could be elevated firing from inhibitory BCs. Surprisingly, the firing rates of APs recorded from the soma of BCs showed no difference between the WT and KO neurons (Supplementary Figure 2a–c). This suggests lack of FMRP may not affect somatic excitability of BCs, but preferentially increase the excitability or downstream Ca^2+^ signaling in their nerve terminals to enhance GABA release. Because the BC terminals were too small to be accessed by patch-clamp electrodes, we manipulated the axonal excitability by changing the membrane potential at their somas. Simultaneous paired recordings from the BC–PN synapses showed that elevated excitability of BC nerve terminals from depolarizing the soma increased the amplitude and frequency of sIPSCs, mimicking the phenotype of *Fmr1*-KO synapses (Supplementary Figure 3a-d).

To test if deletion of *Fmr1* would increase the AP-dependent intra-terminal Ca^2+^ level, we performed multiphoton Ca^2+^ imaging from the boutons of distal nerve terminals by loading a Ca^2+^ indicator (Fluo-4) together with a morphological tracer (Alexa Fluor 594) via patch electrodes into the soma of BCs (Fig. 1i, j). A train of APs delivered at the soma elicited significantly larger area integral of Ca^2+^ transients with a slower decay time course in the *Fmr1*-KO boutons than those in the WT terminals (Fig. 1k–p). These results suggest that the elevated Ca^2+^ level after APs in the nerve terminals underlies the excessive GABA release in the *Fmr1*-KO synapses.

### N-terminus of FMRP interacts with Kv1.2 to regulate excitability and synaptic transmission of BC axonal terminals

**Fig. 2:**
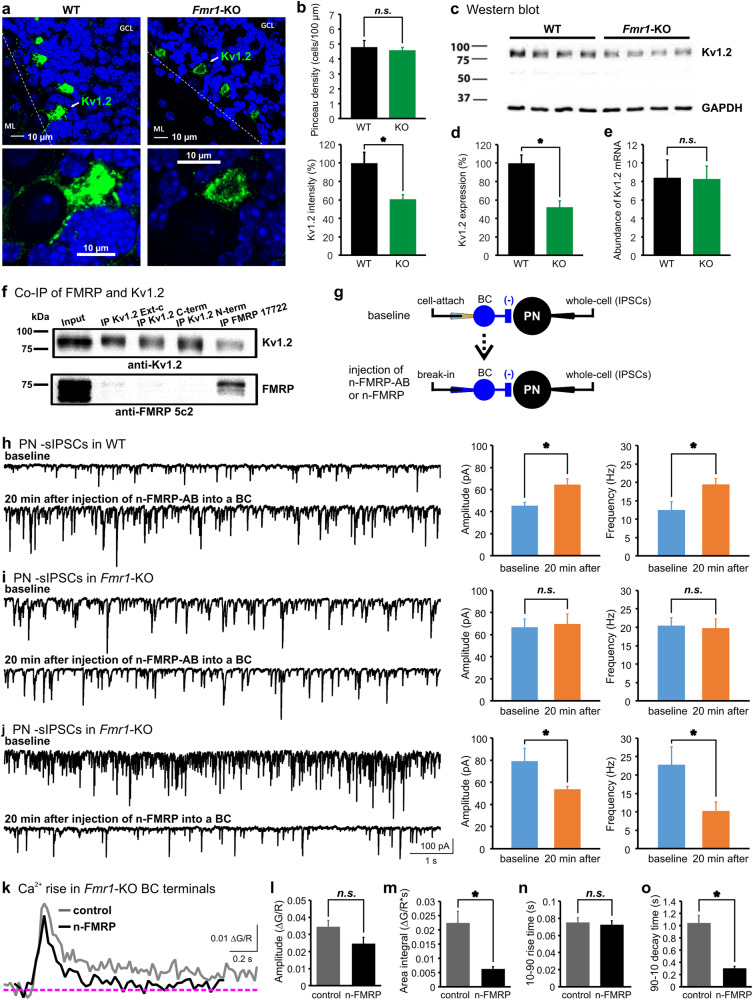
N-terminus of FMRP interacts with Kv1.2 to control inhibitory output at BC nerve terminals. **a** Images of Kv1.2 immunostaining in cerebellar cortex showing reduced expression of Kv1.2 (green) in the *Fmr1*-KO BC axonal terminals. Individual pinceau marked with a single arrow (top) are revealed at a higher magnification (bottom). Dotted lines indicate Purkinje cell layer. ML molecular layer, GCL granule cell layer. **b** Comparison of the density of pinceau (top) and fluorescence intensity (bottom) of Kv1.2 labeling between WT and *Fmr1* KO. **c** Representative western blot of Kv1.2 taken from the whole cerebellar homogenates of WT and KO samples. **d** Analysis of western blot revealing a significant decrease in Kv1.2 expression in KO (*n* = 7) compared to WT (*n* = 8) mice. **e** Quantitative RT-PCR analysis showed no difference in Kv1.2 mRNA abundance between the WT (*n* = 6) and KO (*n* = 6) cerebellum. **f** Co-IP by anti-Kv1.2 and anti-FMRP antibodies, indicating the two molecules interact at the protein–protein level. **g** Schematic depiction of paired recording configurations: top, presynaptic cell-attached mode for baseline measurements; bottom, infusion of N-terminus of FMRP binding antibody (n-FMRP-AB) or N-terminus FMRP fragment (n-FMRP) into the BC terminal until equilibrium. Presynaptic and postsynaptic holding potential was set at −80 and −60 mV, respectively. **h–j** sIPSCs (left panels) recorded from PNs before and 20 min after diffusion of n-FMRP-AB (NBP2-01770, Novus Biologicals, 1:2000 dilution) into a WT (**h**) or KO (**i**) synapse, and n-FMRP (H00002332-P01, Novus Biologicals, 1:100 dilution) into a KO (**j**) synapse. The amplitude and frequency of sIPSCs are summarized in the right panels for the conditions in **h** (*n* = 5), **i** (*n* = 6), and **j** (*n* = 6). **k**–**o** Averaged intra-terminal Ca^2+^ rise from 5 trials evoked by AP trains (100 Hz, 100 ms) delivered to the soma of BCs in KO synapses with (black) or without n-FMRP (control, grey). The amplitude (**l**), area integral (**m**), 10–90 rise time (**n**), and 90–10 decay time (**o**) of Ca^2+^ transients are summarized for the control (grey bars, *n* = 7) and n-FMRP (black bars, *n* = 5) groups

The excitability of BC terminals is controlled by voltage-gated, particularly low-threshold potassium channels, Kv1.1 and Kv1.2 [25–27]. By lowering the spike amplitude and resting potential, these channels constrain Ca^2+^ load into nerve terminals, and accelerate Ca^2+^ clearance by facilitating membrane repolarization after spikes [28]. Given that blockade or mutation of Kv1s increases the presynaptic excitability and GABA release [29, 30], we investigated the status of Kv1s in the *Fmr1*-KO cerebellum by immunocytochemical analysis. We found Kv1.2 immunostaining was most prominent in the BC axon terminals (Fig. 2a). The count of BC pinceau stained with the Kv1.2 antibody was not different between the WT and KO mice, suggesting deletion of *Fmr1* did not alter the gross number of BC terminals (Fig. 2a, b). However, the immunostaining intensity and distribution pattern of Kv1.2 were markedly different. In the WT cerebellum, Kv1.2 channels were highly concentrated in the pinceau and synaptic contacts to the cell body of PNs whereas they appeared more sparse in the KO synapses, leading to a nearly 50% reduction in the fluorescence intensity. Consistently, western blots of cerebellar homogenates revealed a substantial decrease in Kv1.2 protein in the *Fmr1*-KO mice (Fig. 2c, d). In contrast to Kv1.2, the same analysis showed a comparable amount of Kv1.1 protein between *Fmr1*-KO (107.1 ± 15.4%) and WT (100.0 ± 13.3%) cerebellum. Interestingly, reverse transcription polymerase chain reaction (RT-PCR) assay displayed no change in the total Kv1.2 mRNA by the elimination of FMRP (Fig. 2e), indicating a deficit in post-transcriptional regulation of the Kv1.2 level.

Recent studies suggest that FMRP can play a non-canonical role by interacting with other proteins including ion channels [31–34]. We postulated that FMRP might directly modulate Kv1.2 channels, aside from its classical role as a translational repressor [35]. To test this, whole cerebellum from the WT mice were homogenized and subjected to immunoprecipitation (IP; Fig. 2f). As expected, FMRP was highly concentrated after IP by a specific FMRP antibody (ab 17722) in the WT, but not in *Fmr1*-KO cerebellum (Supplementary Figure 4). Once the anti-FMRP IP was then immunoblotted with an antibody against Kv1.2, we found that the two proteins co-immunoprecipitated (co-IP, Fig. 2f). Yet Kv1.2 protein did not co-IP with FMRP in the *Fmr1-*KO mice, confirming that the Kv1.2 IP was through FMRP but not from any other non-specific interaction (Supplementary Figure 4). The antibodies that did not bind to the N-terminus of FMRP (ab 17722 and 5c2) were able to pull down Kv1.2, implying that the N-terminus of FMRP could form protein complexes with Kv1.2. Reversely, when the anti-Kv1.2 antibodies were used, only the Kv1.2 antibody recognizing the extracellular domain (Kv1.2 Ext-c) could immunoprecipitate FMRP under improved conditions (Supplementary Figure 5). A possible explanation is that both FMRP and the intracellular-targeting Kv1.2 antibodies (Kv1.2 C-term and Kv1.2 N-term) compete for sterically hindered binding location on the Kv1.2 intracellular domains.

To investigate the potential roles of the N-terminus of FMRP in regulating GABA release from interneurons, we injected an antibody specifically occluding the N-terminus of FMRP (n-FMRP-AB) into BCs and simultaneously recorded sIPSCs from PNs (Fig. 2g). We found this antibody increased the amplitude and frequency of sIPSCs, acutely converting the WT into KO-like synapse (Fig. 2h). By contrast, n-FMRP-AB had no effect on the KO synapses, indicating its specificity in perturbing the function of FMRP (Fig. 2i). Conversely, when a purified N-terminal FMRP fragment (n-FMRP) was added to the *Fmr1*-KO BCs, it fully rescued the synaptic deficit by normalizing the AP-evoked intra-terminal Ca^2+^ rise (Fig. 2j–o). Collectively, these observations pinpoint that the N-terminus of FMRP interacts with Kv1.2, which is necessary and sufficient to control presynaptic excitability and GABA release.

### C-terminus of Kv1.2 interacts with FMRP in a phosphorylation-dependent manner

**Fig. 3:**
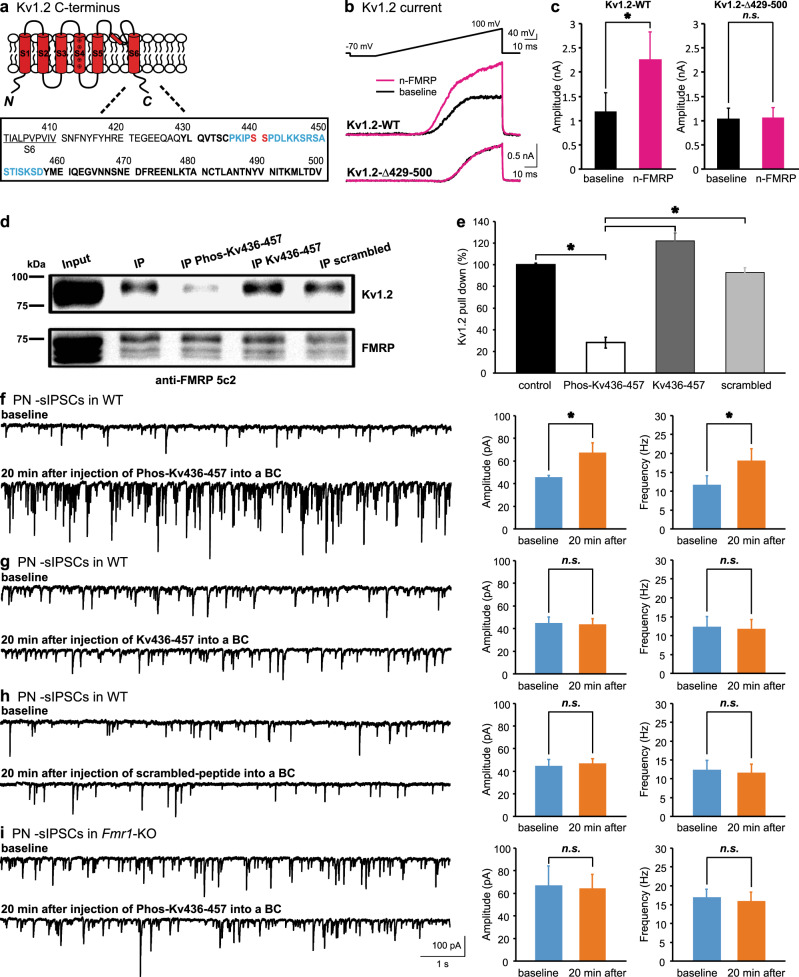
Direct interaction between FMRP and Kv1.2 in a phosphorylation-specific manner. **a** Illustration of an alpha subunit of Kv1.2 channel with extensive details of intracellular C-terminus. **b** K^+^ current generated by a voltage ramp (−90 to 100 mV, top) from CHO cells expressing WT (Kv1.2-WT, middle) or truncated Kv1.2 (Kv1.2-Δ429-500, bottom) before and after infusion of N-terminus FMRP protein (n-FMRP, H00002332-P01, Novus Biologicals, 1:100 dilution). **c** The changes in amplitude of K^+^ current by n-FMRP is summarized for Kv1.2-WT (*n* = 8) and Kv1.2-Δ429-500 (*n* = 8) constructs. **d** Co-IP of Kv1.2 and FMRP in the absence or presence of three peptides targeting the sequence (436–457) of C-terminus of Kv1.2 as highlighted in **a** phosphorylated (PhosKv436-457), non-phosphorylated (Kv436-457), and phosphorylated scrambled peptide. **e** Summary of effect of the three peptides. Note that only PhosKv436-457 significantly reduces the production of Kv1.2 pull down as compared to the control conditions. **f–h** sIPSCs (left panels) recorded from PNs before and 20 min after infusion of the three peptides (2.5 mM) into WT synapses. The changes in the amplitude and frequency of sIPSCs are summarized in the right panels for PhosKv436-457 (*n* = 6, **f**), non-phosphorylated Kv436-457 (*n* = 6, **g**), and scrambled (*n* = 5, **h**) peptides. Consistent with the co-IP experiment, only PhosKv436-457 increases the amplitude and frequency of sIPSCs, acutely imparting a WT synapse with the KO phenotype. **i** Same quantifications for injecting PhosKv436-457 (2.5 mM) into KO synapses (*n* = 5) showing no effect on sIPSCs

The Kv1.2–FMRP interaction appeared to necessitate phosphorylation, as the co-IP experiments required phosphatase inhibitors [31]. Additionally, the antibody against the C-terminus of Kv1.2 could not co-IP FMRP (Fig. 2f), suggesting that the anti-Kv1.2 antibody and FMRP could compete for an overlapping, and sterically hindered, binding site at the C-terminus of Kv1.2, where several serine residues were known to be phosphorylated (Fig. 3a) [36]. To test this, we transfected Chinese hamster ovary (CHO) cells with a WT or mutant Kv1.2 (Kv1.2-Δ429-500) construct in which the C-terminus after amino acid 429 had been truncated. When we made whole-cell recordings from positively transfected cells (with GFP as a marker), we found that both the WT and mutant Kv1.2 channels were able to conduct currents evoked by voltage ramps (Fig. 3b, c). However, when the n-FMRP fragment was included in the intracellular solution, the ramp currents from cells expressing the WT Kv1.2 gradually increased in amplitude and reached maximum in about 5 min, with a notable leftward shift in their voltage-dependence and a lower activation threshold. In contrast, the same paradigm yielded no significant alterations in cells expressing the truncated Kv1.2. These results indicate that FMRP enhances the gating and current density of Kv1.2 channels by directly interacting with their C-terminus.

To search for the motif for Kv1.2 to interact with FMRP, we designed a series of peptides covering the sequence of the Kv1.2 C-terminus. Among them, we found that a Kv1.2 peptide, which encompassed two tandem phosphorylation sites at serine 440 and serine 441 (PhosKv436-457, ^436^PKIP**p**S**p**SPDLKKSRSASTISKSD^457^) in the doubly-phosphorylated state, greatly lowered the amount of Kv1.2 (by 75%) that was co-IP with FMRP from cerebellar homogenates (Fig. 3d, e). Neither the non-phosphorylated peptide with the identical sequence (Kv436-457, ^436^PKIPSSPDLKKSRSASTISKSD^457^) nor the doubly-phosphorylated scrambled peptide (IKPSTLSDSRAK**p**SSDP**p**SKPSIK) inhibited the Kv1.2–FMRP binding activity. Similarly, PhosKv436-457 impeded the protein binding in the reverse co-IP, where the Kv1.2 antibody (Kv1.2 Ext-c) pulled down FMRP (Supplementary Figure 5). These results indicate that phosphorylation of the serine sites is critical for the sequence-specific interaction.

When each of the peptides were injected into the WT BCs, we found that only the phosphorylated PhosKv436-457, but not the non-phosphorylated or scrambled phosphorylated peptide, significantly elevated the amplitude and frequency of sIPSCs obtained from PNs (Fig. 3f–h). Furthermore, PhosKv436-457 did not affect GABA release in the *Fmr1*-KO synapses, confirming its specific interference of the FMRP–Kv1.2 complex (Fig. 3i). These data suggest that a fraction of endogenous Kv1.2 channels may be phosphorylated to bind FMRP, and that the phosphorylated Kv1.2 peptide can compete for this interaction. Such a phosphorylation-dependent interaction provides the interneuron nerve terminals with an activity-dependent mechanism to regulate the magnitude of Kv1.2 currents and dynamically fine-tune the inhibition onto principal neurons.

### Phosphorylated Kv1.2 directly binds FMRP in a dimer–dimer configuration

**Fig. 4:**
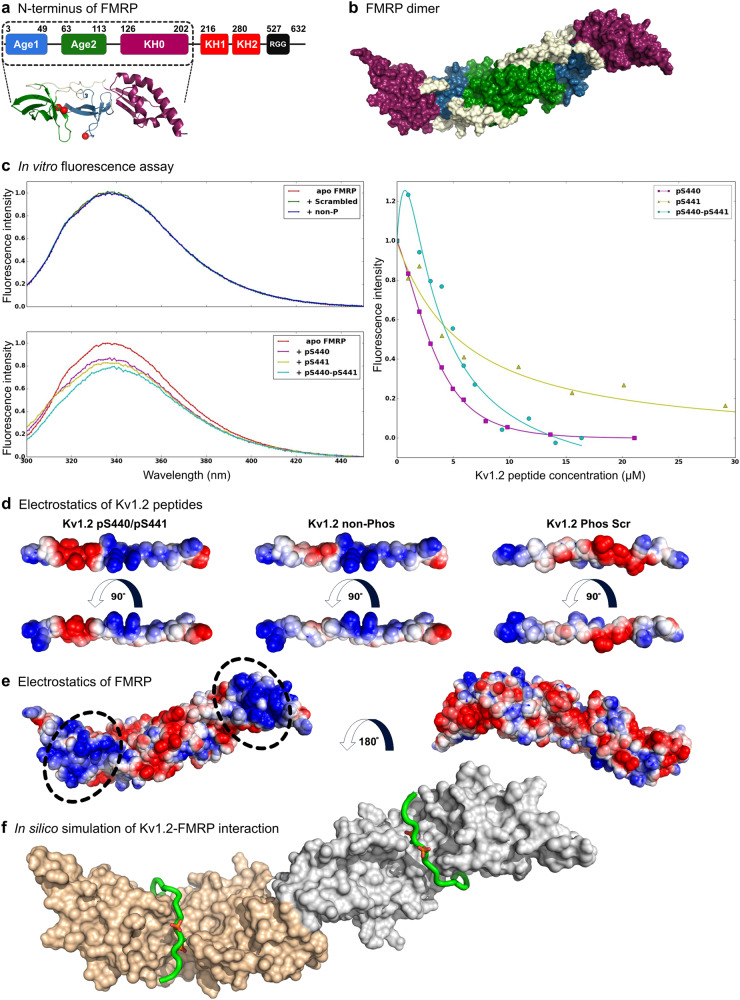
Molecular complex of FMRP and Kv1.2. **a** Schematics of human FMRP structure showing tandem Agenet (Age1 and Age2) domains followed by three K Homology (KH) domains (KH0, KH1, and KH2) before ending with a long C-terminal intrinsically disordered region containing an RGG box. The cartoon representation of the crystal structure of Age1–Age2–KH0 (enclosed by dashed lines) with the red spheres showing the location of tryptophan residue. **b** The Age1–Age2–KH0 construct solved as an Age2-based dimer (PDB ID code 4OVA). **c** Left top panel shows the normalized fluorescence emission spectrum of apo-FMRP (red) and FMRP mixed with non-phosphorylated Kv1.2 C-terminal peptide (blue) or doubly-phosphorylated scrambled peptide (green). Lack of spectral changes indicates no binding. Bottom panel shows the spectral changes observed between apo-FMRP (red) and FMRP mixed with pS440 (purple), pS441 (yellow), or pS440-pS441 (cyan), all indicating a phosphorylation-mediated interaction (see text for details). Right panel shows binding profiles of pS440 (purple), pS441 (yellow), and pS440/pS441 (cyan) fitted with an FMRP dimer binding two-peptide molecules with cooperativity (for pS440) and without cooperativity (for pS441 and pS440/pS441). The binding parameters and *χ*^2^ statistics for fitting the different peptides to FMRP are given in Supplementary Table 1. **d** Electrostatic profiles for doubly-phosphorylated (left), non-phosphorylated (middle), and doubly-phosphorylated scrambled (right) Kv1.2 peptides. **e** Electrostatic profiles for the Age1–Age2–KH0 dimer. **f** A representative docked model of the FMRP (surface):Kv1.2 pS440/pS441 peptide (ribbon) with the phosphorylated serines shown as orange and red sticks

To validate our phosphorylation-dependent interaction model, we generated a recombinant full-length FMRP (isoform 1) and tested its interaction with the Kv1.2 peptides by measuring intrinsic protein fluorescence. FMRP has 5 tryptophan (indicated as red spheres) and 14 phenylalanine residues distributed throughout the protein (Fig. 4a), while the Kv1.2 peptides lack fluorescent aromatic amino acids (F, Y, or W), making in vitro fluorescence spectroscopy a valuable technique to detect direct binding and/or conformational changes associated with their interaction. We found no change in the fluorescence emission spectrum of FMRP with the non-phosphorylated and scrambled peptides (Fig. 4c). In contrast, significant spectral changes were observed with addition of the singly (pS440, pS441) or doubly (pS440/pS441, i.e., PhosKv436-457) phosphorylated peptides in a concentration-dependent manner, reinforcing the direct and specific interaction between the phosphorylated C-terminus of Kv1.2 and FMRP.

It has been shown that the N-terminal Age1–Age2–KH0 domains of FMRP form Age2 domain-based dimers (Fig. 4b) [37]. Therefore, we examined the isotherm binding data by fitting to different binding models to determine the likely oligomeric state of the protein and the stoichiometry of binding [38]. Our analyses suggested that the model in which FMRP existed as a dimer gave the best fit for the pS440 peptide (Supplementary Figure 6). Because Kv channels are composed of tetramers of homo- or heteromeric dimers, it can be rationalized that two C-termini of Kv1.2 subunits directly bind to one FMRP dimer. Intriguingly, in this model pS440 binds dimeric FMRP in a co-operative mechanism with dissociation constants *K*_D1_ and *K*_D2_ of 33.9 ± 6.2 μM and 0.42 ± 0.08 μM, respectively, denoting that the binding affinity of the second ligand is synergistically enhanced ~80 folds by the binding of the first ligand (Supplementary Table 1). However, pS441 and pS440/pS441 did not produce co-operative binding effects, but they did bind with similar affinities. Taken together, these data implicate multiple levels of dynamic interactions between FMRP and Kv1.2 in a phosphorylation-dependent manner.

Next, we performed an in silico docking of the doubly-phosphorylated Kv1.2 peptide, pS440/pS441, onto the dimeric FMRP N-terminal domain (Age1–Age2–KH0) crystal structure (PDB ID code 4OVA) [37]. We first analyzed geometric (groove and ridges) and/or physico-chemical (electrostatic and hydrophobic) complementarities between the two structures as a potential binding interface. The Kv1.2 peptide ^436^P**K**IPSSPDL**KK**S**R**SASTIS**K**SD^457^ was highly basic, due to the presence of four lysine and one arginine residues, which were distributed along the primary structure of the peptide. However, an intense patch of negative charge in the phosphorylated peptide, presented by the doubly charged phosphate groups (PO_4_^2−^) of pS^440^ and pS^441^ and singly charged carboxylate group (CO_2_^−^) of D^443^, was formed (Fig. 4d). Thus phosphorylation generated an alternating positive–negative–positive charge pattern carried by ^437^K, (^440^pS/^441^pS/^443^D), and ^445^K^446^K, respectively. The pattern was absent in the non-phosphorylated and doubly-phosphorylated scrambled peptides, potentially explaining their inability to bind with FMRP.

The structure of FMRP also possessed interesting features that could facilitate the protein–protein binding. Myrick et al. [39] described a continuous stretch of positive surface formed by the Age1 and KH0, with a small basic groove between Age1 and Age2, next to an acidic surface formed by Age2 (Fig. 4e). The complementary electrostatic pattern of the phosphorylated Kv1.2 peptide and the basic groove surface of FMRP that could fit the acidic PpSpSP motif of Kv1.2 suggested a mechanism that could facilitate the protein binding. To visualize such an interaction, we performed an electrostatic-based docking of the extended doubly-phosphorylated Kv1.2 peptide to the FMRP N-terminal Age1–Age2–KH0 domains, yielding highly stable modes of engagements based on distinct energy terms (Fig. 4f and Supplementary Figure 7). These simulations provide structural insights into specific grooves on the Tudor and KH0 domains of FMRP that may enable its optimal interaction with the phosphorylated C-terminal motif of Kv1.2.

### Kv1.2 agonist alleviates inhibitory overtone in the cerebellum and normalizes elevated startle response and impaired sociability in the *Fmr1*-KO mice

**Fig. 5:**
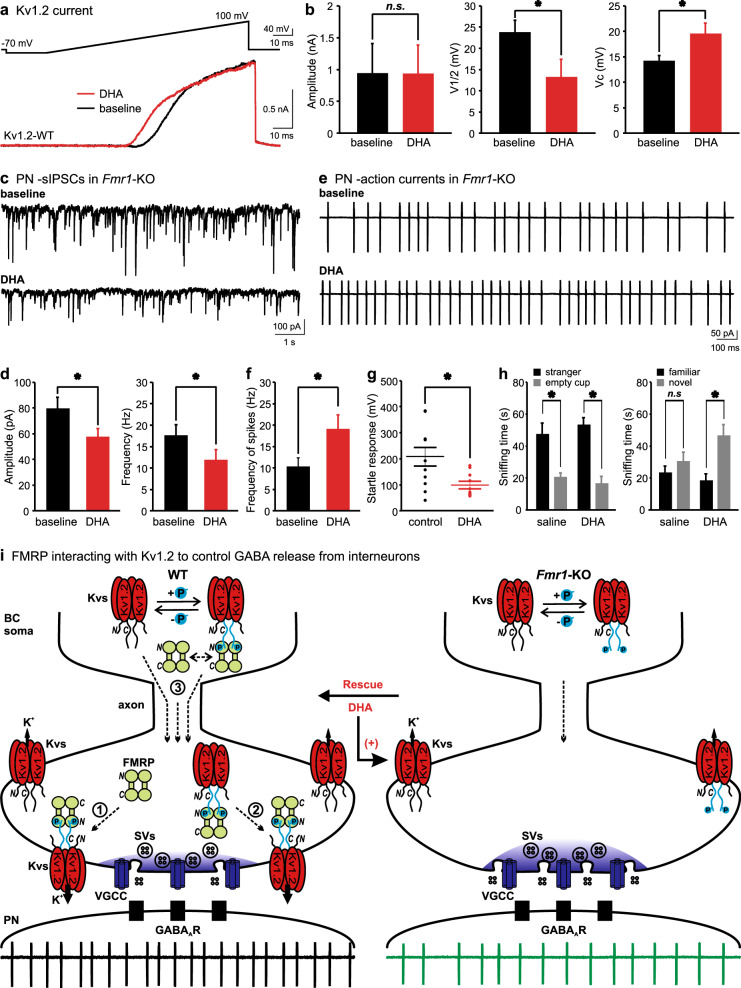
DHA rescue and graphical overview of the function of FMRP at the BC–PN synapses. **a**, **b** Currents generated by a voltage ramp (−90 to 100 mV) from CHO cells transfected with recombinant Kv1.2 in the absence (black) or presence (red) of DHA (1 µM). These currents are fit with a Boltzmann function: *f*(V) = Imax/(1+e^(V1/2−V)/Vc^)+C, in which “Imax” denotes the theoretical value of the maximal amplitude of Kv1.2 currents; “V1/2” indicates the depolarization voltage needed to produce the half-maximal amplitude of the currents; “Vc” is the slope factor describing the steepness of the Boltzmann curve. The parameters for the two groups (*n* = 7) are summarized in (**b**). **c**, **d** sIPSCs recorded from PNs in *Fmr1*-KO slices with (bottom) or without (top) DHA (10 µM). Changes in the amplitude and frequency of sIPSC (*n* = 8) are quantified in (**d**). **e**, **f** APs from PNs before (top) and after (bottom) DHA application (10 µM). Note: DHA increases the frequency of APs in the *Fmr1*-KO synapses (**f**, *n* = 7). **g** Startle responses to a sound stimulation (120 dB) from regular (*n* = 10) or DHA-feeding (*n* = 11) *Fmr1*-KO mice. **h** Sniffing time spent on the stranger and empty cup in the sociability session (left); and spent on the novel and familiar mouse in the social novelty session (right) of the three-chambered test by *Fmr1*-KO mice injected with DHA (200 mg/kg, *n* = 7) or saline (*n* = 7). **i** Schematics of the BC-PN synapse in WT (left) and *Fmr1*-KO (right) cerebellum. In WT BCs, there are two populations of Kv1.2 channels being in either non-phosphorylated or phosphorylated state (with C-termini colored in black and turquoise respectively; +/−P to symbolize phosphate modification). The N-terminus of FMRP dimers directly interact with C-terminus of Kv1.2 only in a phosphorylated state. This interaction enhances the gating (Pathway 1), membrane targeting (Pathway 2), and/or axonal trafficking (Pathway 3) of Kv1.2 in the nerve terminal to control its excitability and Ca^2+^ dynamics (blue) critical for tuning inhibitory neurotransmission and ensuring the final output from principal neurons (i.e., PNs). In *Fmr1*-KO BCs, loss of FMRP downregulates the expression and function of Kv1.2, thereby elevating presynaptic excitability and Ca^2+^ level, increasing GABA release and reducing PN firing activity. As an allosteric agonist, DHA (arrow) acts on the existing Kv1.2 channels at the BC terminal to suppress the presynaptic excitability and excessive GABA release, rescuing the low firing phenotype of PNs in the *Fmr1*-KO cerebellum

Knowing that lack of FMRP lowers the expression and activity of Kv1.2, we reasoned that enhancing the function of Kv1.2 channels at the BC terminals might restate the normal inhibition in the cerebellum. Therefore, we screened for Kv1.2 agonists using CHO cells expressing Kv1.2. We found DHA (1 µM), an omega-3 fatty acid, produced a significant leftward shift in the Kv1.2 currents evoked by a voltage ramp (Fig. 5a) with little effect on their amplitude, in line with previously reported effect of polyunsaturated fatty acids on recombinant Shaker K^+^ channels [40, 41]. By fitting the currents with a Boltzmann function, we revealed that DHA decreased the half-activation voltage (V_1/2_) and increased the slope factor (Vc), while the maximal amplitude was unaffected (Fig. 5b). This suggests DHA functions as a positive allosteric modulator of Kv1.2 channels, likely by interacting with the voltage-sensor to lower the activation threshold [40, 41]. DHA is particularly effective in the critical range of membrane potentials for dampening the membrane excitability, similar to the effect of FMRP on Kv1.2 channels (Fig. 3b).

To test if DHA played a role in inhibitory neurotransmission, we applied DHA (10 µM) to cerebellar slices taken from the *Fmr1*-KO animals. DHA clamped excessive GABA release, as demonstrated by the reduced amplitude and frequency of sIPSCs (Fig. 5c, d). When the cerebellar output was examined by recording APs from PNs in the cell-attached mode, without perturbing intracellular Cl^−^ homeostasis, we found DHA enhanced the spike frequency of PNs, restoring the firing rate of *Fmr1*-KOs to the same level as the WTs (Fig. 5e, f). To investigate the effect of DHA on behaviors of the *Fmr1-KO* mice, we fed them with DHA enriched rodent-chow (0.5% w/w) for 1 week before measuring their acoustic startle reflex (ASR), in which the vermis of the cerebellum plays an indispensable role in gating sensorimotor reactions [42]. ASR is one of the few objective indices that show consistent impairments in human and mouse models of FXS [43, 44]. DHA can cross blood–brain barrier, as shown previously [45]. Our double-blind experiments demonstrated that DHA significantly attenuated ASR from a 120-dB sound stimulation in adult *Fmr1-KO* mice, as compared to the *Fmr1*-KO control group without DHA supplement (Fig. 5g). Furthermore, we administered DHA by intraperitoneal injection in *Fmr1*-KO mice and subjected them to the three-chambered sociability test [46, 47]. Compared to the saline group, DHA-treated animals queried the stranger more than familiar mouse, indicating that DHA improved the deficient social novelty recognition of these mice (Fig. 5h) [48, 49]. Yet, the same application of DHA did not alter the social behaviors of WT animals (Supplementary Figure 8). Although the BC terminals express the highest level of Kv1.2 in the entire brain [25, 50], systematic administration of DHA could ameliorate the FXS impairments by influencing other brain regions and other ion channels as well [51]. Despite of no observable effects of DHA in WT mice, additional experiments using cell-specific deletion of *Fmr1* or Kv1.2 are needed to exclude potential off-target actions of DHA. Collectively, these results suggest that by boosting the function of Kv1.2, DHA effectively controls GABA release and rectifies the firing deficits of postsynaptic neurons in vitro as well as normalizes the startle response and sociability of the *Fmr1*-KO mice in vivo.

## Discussion

Using the cerebellar BC–PN synapse as a model, we show that deletion of *Fmr1* leads to excessive GABA release from BCs to suppress the firing activity of PNs. This inhibitory overtone results from increased Ca^2+^ transients and excitability due to a lower level of Kv1.2 expression in the interneuron axonal terminals. We further map out a serine-rich motif in the C-terminus of Kv1.2, which enables its direct phosphorylation-specific interaction with the N-terminus of FMRP. Finally, we provide compelling evidence that increasing the activity of Kv1.2 with DHA fully restores the synaptic inhibition and certain behavioral phenotypes in the FXS mouse model (Fig. 5i).

Studies from different animal models, including *Tsc1*, *Shank 2*, and *PTEN* KO mice, suggest that attenuated firing of PNs is a recurring denominator for autism [10, 52, 53]. Despite recapitulating the phenotype of these mutations, the impaired activity of PNs in *Fmr1*-KO mice is mediated exclusively by presynaptic over-inhibition from interneurons, independent of any changes in postsynaptic GABA_A_ receptors or intrinsic excitability of PNs.

FMRP regulates the expression and function of several ion channels [33, 34, 54–56]. Our study demonstrates that FMRP can directly bind phosphorylated Kv1.2 to control presynaptic excitability, either by enhancing the gating (Pathway 1), surface expression (Pathway 2), and/or axonal trafficking of Kv1.2 channels (Pathway 3) at the nerve terminal (Fig. 5i). Acute application of n-FMRP is unlikely to affect Kv1.2 de novo protein translation. Our evidence from CHO cells expressing Kv1.2 channels support such a conceptual model, where FMRP increased the amplitude and left-shifted the voltage-dependence of the macroscopic currents (Fig. 3b, c). These may be resulted from an insertion of new Kv1.2 channels onto the membrane and/or enhancing gating of the existing channels (Pathways 1 & 2). Injection of FMRP in the soma of BCs toned down the Ca^2+^ transients and excessive GABA release from BC terminals in the *Fmr1*-KO synapses (Fig. 2j–o) suggests the potential engagement of FMRP in axonal trafficking of Kv1.2 (Pathway 3). Rescuing effects of n-FMRP in the *Fmr1*-KO synapses implicates that FMRP readily binds to phosphorylated Kv1.2 channels to suppress the excitability of BC terminals via these pathways. Additional evidence from our experiments that n-FMRP can normalize synaptic deficits in the *Fmr1*-KO slices pretreated with a protein synthesis inhibitor anisomycin (data not shown) further supports our notion that the non-canonical and pleiotropic roles of FMRP may be a key missing link for targeting, gating, and trafficking of Kv1.2 channels to the central nerve terminals beyond its traditional roles as a mRNA translational repressor.

Knowing that Kv1.2 are particularly abundant in interneurons, under-expression of these channels is likely a common feature of presynaptic dysregulation of inhibition, and may contribute to a variety of pathological phenotypes associated with FXS, autism, and seizure which are exhibited by Kv1.2 mutant mice or polymorphisms observed in humans [30, 57]. Kv1.2 is also expressed at the presynaptic site of fast-spiking sensory neurons in the auditory brainstem and excitatory neurons in the cortex [58, 59]. Deletion of FMRP may change the channel function and neural firing, particularly during high-frequency activity [28], thereby promoting glutamate release that could ultimately exacerbate the E/I imbalance. Given that FMRP and phosphorylation signaling strongly regulate homeostatic and activity-dependent synaptic plasticity, we suggest that these interactions diversify the options for presynaptic nerve terminals to fine-tune neurotransmission with much faster dynamics than that required for de novo translation.

Because Kv1.2 channels are downregulated but not absent at the *Fmr1*-KO BC terminals, we have strategically targeted the remaining Kv1.2 with DHA to rescue the synaptic and behavioral deficits in the *Fmr1*-KO mice. Due to the abundance of Kv1.2 in these nerve terminals, we interpret that DHA preferentially inhibits the excitability of BC terminals rather than that of PNs. Otherwise, DHA would have reduced the excitability and firing rate of PNs if it primarily acted on postsynaptic K^+^ conductance. This insight may help explain the long-standing controversy on the clinical benefit of omega-3 fatty acids, including DHA (or fish oil), to treat autism [60–62], highlighting the importance of an in-depth understanding of the mechanistic heterogeneity underpinning autism for personalized medicine.

As the only output from the cerebellar cortex, lower activity of PNs disinhibits downstream neurons in the deep cerebellar nuclei, which gate the outgoing information to the thalamus and prefrontal cortex, potentially influencing the development of motor, cognitive, and affective functions. Our results not only provide molecular insights into the novel role of FMRP in tuning presynaptic inhibition, but also identify a loss of the Kv1.2–FMRP interaction as a key locus for the pathogenesis of FXS. Experiments with DHA provide a proof of principle that the molecular locus can be a valid target for therapeutic intervention and a strong candidate for clinical trials on FXS. Given that FMRP is also reduced in other forms of autism [63], further investigating this novel mechanism will help strategize the development of n-FMRP gene fragment and Kv1.2-specific agonists for gene and pharmacological therapies to restore the E/I balance in neuropsychiatric disorders.

## Materials and methods

### Animal models

All mice experiments were in accordance with the guidelines by the Canadian Council on Animal Care and US Department of Agriculture Animal Welfare Act and Animal Welfare Regulations; and approved by the Hospital for Sick Children and University of Toronto Animal Care Committees and Institutional Animal Care and Use Committee, University of Minnesota. Wild-type (WT) C57BL/6 and *Fmr1* knockout (KO) mice (backcrossed >10 generations on the C57BL/6 background) were purchased from the Jackson Laboratory (donated by Dr. William Greenough, University of Illinois). Both genders were used unless otherwise specified. Mice at postnatal days 26–32 were subject to electrophysiological and other analyses unless otherwise specified.

### Cerebellar slice preparation

Parasagittal cerebellar slices were sectioned at a thickness of 300 μm using a vibratome (VT 1200S, Leica) in ice-cold modified artificial cerebral spinal fluid (ACSF) containing (in mM): sucrose (218), KCl (3), glucose (10), NaH_2_PO_4_ (2.5), NaHCO_3_ (26), MgCl_2_ (2), and CaCl_2_ (2) continuously bubbled in 95% O_2_ and 5% CO_2_ with a final pH of 7.3. Subsequently, slices were incubated in oxygenated standard ACSF including (in mM): NaCl (125), KCl (2.5), glucose (10), NaH_2_PO_4_ (1.25), sodium pyruvate (2), myo-inositol (3), ascorbic acid (0.5), NaHCO_3_ (26), MgCl_2_ (1), and CaCl_2_ (2) at 37 °C for 30 min prior to experimentation.

### CHO cells expressing Kv1.2

CHO cells (ATCC CCL-61) were maintained in 60 mm tissue culture dishes (Nunc, Thermo Fisher Scientific) containing 5 ml of Ham’s F12K media (Wisent) supplemented with 10% fetal bovine serum and 1% penicillin–streptomycin (Wisent); and grown at 37 °C, 5% CO_2_. Cells were split twice weekly to prevent overgrowth by removing the culture medium, washing the cells once with 10 ml phosphate-buffered saline (PBS) (Wisent) and then incubating with 1 ml Trypsin EDTA (0.25%) for 3 min at room temperature. Subsequently, 5 ml of culture medium was added to the dish, and cells were dissociated mechanically. Then 0.5 ml of medium containing dissociated cells (**~**2 × 10^5^ cells) was transferred to a new dish. CHO cells (1 × 10^4^ cells/cm^2^) were plated 1 day before transfection onto coverslips. Transfection was carried out using the FuGene HD (Roche) method with Kv1.2-GFP (gift from Drs. Gang Xie and John Roder, University of Toronto). After transfection, cells were maintained in Ham’s F12K media for 48 h before experiments.

### Electrophysiology

Brain slices were transferred to a recording chamber mounted on an Olympus microscope with Nomarski optics and a 60× water immersion objective. Slices were continuously perfused with the standard ACSF at a rate of approximately 1 ml/min with supplement of NBQX (10 µM) or bicuculline (10 µM) to block excitatory or inhibitory inputs, respectively. PNs and BCs were identified by their size and location. Patch electrodes had resistances of 2.5–3 and 4.5–6 MΩ for PNs and BCs, respectively. Spontaneous and miniature EPSC and IPSCs from PNs were recorded in the whole-cell voltage-clamp mode at −60 mV. Tetrodotoxin (TTX, 1 μM) eliminated spontaneous spike firing to isolate miniature IPSCs. To record EPSCs, the pipette solution contained (in mM): K-gluconate (97.5), CsCl (32.5), EGTA (5), HEPES (10), MgCl_2_ (1), TEA (30), and lidocaine N*-*ethyl bromide (3) (pH 7.3). To record IPSCs, the pipette solution contained (in mM): K-gluconate (50), CsCl (80), EGTA (5), HEPES (10), MgCl_2_ (1), TEA (30), and lidocaine N-ethyl bromide (3) (pH 7.3). Spontaneous APs at the soma were recorded in the cell-attached mode at a holding potential of −60 mV for PNs and −80 mV for BCs, which manifested as compound inward–outward currents. In this case, the intracellular solution included (in mM): K-gluconate (97.5), KCl (32.5), EGTA (0.1), HEPES (40), MgCl_2_ (1), ATP (2), GTP (0.5) (pH 7.3). The same solution was used to deliver n-FMRP-AB (NBP2-01770, 1:2000 dilution, Novus Biologicals), n-FMRP (H00002332-P01, 1:100 dilution, Novus Biologicals), phos-Kv436-457 (PKIP**p**S**p**SPDLKKSRSASTISKSD, 2.5 mM, Canada Peptide), non-phosphorylated peptide (PKIPSSPDLKKSRSASTISKSD, 2.5 mM, Canada Peptide) and bi-phosphorylated scrambled peptide (IKPSTLSDSRAK**p**SSDP**p**SKPSIK, 2.5 mM, Canada Peptide).

CHO cells transfected with Kv1.2 were positively identified with green fluorescence illuminated briefly by a mercury burner (Olympus). Recordings from CHO cells were made in the extracellular solution containing (in mM): NaCl (140), KCl (2.5), CaCl_2_ (1.3), HEPES (10), glucose (33), pH 7.3. The intracellular solution was the same as used for cell-attached recordings. The holding potential was set at −90 mV and series resistance was 3–6 MΩ, compensated to 90%.

All the electrophysiological experiments were performed at room temperature (~22 °C). All the recordings were acquired on-line, filtered at 4 kHz, digitized at 50 kHz with a dual-channel amplifier (MultiClamp 700A, Molecular Devices) and digitizer (Digidata 1322A, Molecular Devices). Data were analyzed off-line with pClamp 9 (Molecular Devices), MiniAnalysis (Synaptosoft), and Excel 2016 (Microsoft). Reagents were from Sigma (St. Louis, MO), Tocris Cookson (Bristol, UK), and Alomone Labs (Jerusalem, Israel).

### Two-photon Ca^2+^ imaging

Calcium imaging was performed using 810 nm pulsed laser light generated by Chameleon Ultra Ti:Sapphire laser system (Verdi and VPUF laser head; Coherent) and two-photon laser scanning microscope (TPLSM, LSM 710 NLO; Carl Zeiss). The laser beam was scanned across the sample through a 63× water-immersion objective (1.0 NA, W Plan-Apochromat, Carl Zeiss). For recording AP-evoked Ca^2+^ transients, the pipette (4.5–6 MΩ) was filled with (in mM) K-gluconate (97.5), KCl (32.5), HEPES (40), MgCl_2_ (1), Na-ATP (2), Na-GTP (0.5), phosphocreatine di(tris) salt (12), Fluo-4 (0.15; Kd = 345 nM; Invitrogen) and Alexa Fluor 594 hydrazide (0.03; Invitrogen), pH adjusted to 7.3 with KOH (osmolality = 330 mOsm). The extracellular solution was the standard ACSF supplemented with NBQX (10 µM), APV (50 µM), and bicuculline (10 µM) to block excitatory and inhibitory inputs. BCs were clamped at −80 mV and dialyzed for >20 min. APs were generated in the current-clamp mode by current injection (2000 pA in amplitude, 0.2 ms in duration, 100 Hz for 100 ms). The emitted green and red fluorescence signals were spectrally separated using appropriate combinations of filters (BP500-550, BP600-660) and a dichroic mirror (MBS 760+) and detected by non-descanned detectors (NDD). Excitation laser light was kept as low as required to attain a sufficient signal-to-noise ratio and minimal photo-damage. We used frame scan mode (30–60 Hz) for AP train-evoked Ca^2+^ transients. To obtain a sufficient signal-to-noise ratio, 5–10 sweeps were averaged. Imaging data were analyzed using Zen 2009 (Carl Zeiss), Excel, Igor Pro Neuromatic package, and ImageJ software (https://imagej.nih.gov/ij/). Both green and red pixel intensities within a polygonal region of interest were averaged for each frame. To avoid problems with background correction [64] due to the proximity of seal formation and the site of imaging, we carefully prevented dye ejection into the extracellular space (fast sealing, only slightly increased pipette pressure during seal formation). Recordings with increased background fluorescence were excluded from the study. Δ*G* over *R* ratio (Δ*G*/*R*) was calculated for each frame to follow changes in intracellular Ca^2+^ and was defined as (*G* − *G*_basal_)/*R*_basal_, where *G* was the actual average fluorescence of a spatial extent of a nerve terminal, and *G*_basal_ and *R*_basal_ were the baseline fluorescence in the green and red channel measured 50–100 ms before the stimuli. The amplitude of Ca^2+^ transient was the peak of Δ*G*/*R* signal. The rise/decay time of Ca^2+^ transients was estimated by the time required for Ca^2+^ rise/decay from 10/90 to 90/10 percentage of their amplitude. In most cases, one nerve terminal was recorded from a single BC to avoid overrepresentation of a single synapse in the statistics. The diffusion distance, loading time of the dyes, and access resistance of the delivery pipettes on BCs were carefully controlled when comparing the WT and *Fmr1*-KO groups. In Fig. 2k–o, older mice at postnatal days 40–42 were used to show the effect of n-FMRP on the intracellular Ca^2+^ signals.

### Immunocytochemistry

WT and *Fmr1*-KO mice were anaesthetized with ketamine/xylazine and intracardially perfused with 0.1 M PBS followed by 4% paraformaldehyde. Brains were removed and post-fixed overnight in 4% paraformaldehyde at 4 °C. They were subsequently rinsed with PBS and sunk in 30% sucrose/PBS overnight at 4 °C. The cerebellum was removed, embedded in Optimum Cutting Temperature formulation (OCT; Sakura), and sectioned with a cryostat. Floating coronal cerebellar sections (25 μm) were rinsed in PBS then blocked for 1 h at room temperature in PBS containing 5% goat serum and 0.2% Triton X-100. After three 5-min washes, the sections were incubated in primary antibody overnight at 4 °C. Sections were washed 5 × 10 min in PBS then incubated in secondary antibody for 2 h at room temperature. After 5 × 10 min washes, sections were mounted on glass slides with Prolong Gold Antifade (Invitrogen). For sections treated with anti-FMRP (5c2; BioLegend) and anti-Kv1.2 (clone K14/16, 1:500; NeuroMab), antigen retrieval was performed as described previously [65]. Briefly, sections were treated with 0.8% sodium borohydride, washed with PBS then incubated for 45 min at 75 °C in 0.1 M Na citrate, pH 6.0. After cooling to room temperature, sections were rinsed with PBS, blocked and treated with primary and secondary antibodies as described above. The secondary antibodies were goat anti-mouse Dylight 488 (1:2000; Jackson Laboratories) and goat anti-rabbit Dylight 549 (1:1000; Jackson Laboratories).

For quantitation, digital images of the region of interest (ROI) were captured using a Hamamatsu ORCA 285 CCD camera mounted on a Nikon E1000 microscope at 10× or 20× magnification with identical exposure times for all sections within each experiment. In each experiment, a total of 6–12 images were captured from 3–6 sections of each brain. Staining intensities were analyzed using ImageJ software (NIH). For each image, a ROI was drawn and the Mean Grey Value (sum of grey values divided by total number of pixels) was measured. The Mean Grey Values for all images were averaged for each genotype and expressed as a percentage of the WT value.

### Western blotting

Quantitative western blotting was performed as previously described [66]. Briefly, WT and *Fmr1* KO mice were euthanized by cervical dislocation and the brains were removed and placed on ice. The cerebellum was isolated and homogenized in ice-cold 50 mM Tris–HCl, 1% SDS, pH 7.4, supplemented with protease inhibitor cocktail (Roche) using a Potter-Elvehjem grinding chamber. The protein concentration was determined using the BCA assay (Sigma). Equal amounts of protein (10 µg) were loaded onto a 10% polyacrylamide-SDS gel and transferred onto a nitrocellulose membrane after electrophoresis. The membranes were blocked in 5% milk for 1 h and incubated at 4 °C overnight with one of the following primary antibodies: mouse monoclonal anti-Kv1.2 (clone K14/16; 1:1000; NeuroMab); mouse anti-GAPDH antibody (GAPDH-71.1; 1:80,000; Sigma-Aldrich). After washing, a goat anti-mouse or goat anti-rabbit (Jackson Labs) HRP-conjugated secondary antibody was applied for 2 h. The immunoreactive proteins were visualized using the FluorChem^TM^ MultiImage Light Cabinet (Alpha Innotech). Densitometric analysis was carried out using the AlphaEaseFC software (Alpha Innotech). The intensity of the band of interest was normalized relative to the GAPDH band intensity and protein expression in WT and *Fmr*1 KO animals was presented as a percentage of WT expression levels.

### Quantitation of Kv1.2 mRNA

Whole cerebellums were dissected from WT and *Fmr1* KO mice and stored in RNA save (FroggaBio Scientific Solutions) at 4 °C for 2–7 days. Total RNA was extracted from tissues using the RNeasy Plus Mini Kit (Qiagen) and 2 µg of total RNA was reverse transcribed with random nonamers (Sigma) using the Superscript II kit (Invitrogen) following the manufacturer’s instructions. cDNA was diluted 1 in 25 and quantitative RT-PCR was performed using an ABI Prism 7900 HT (Applied Biosystems) using the Sybr Green detection system. Five microliters of diluted cDNA was used as a template for real-time PCR quantification. Each sample was evaluated for Kv1.2 and a housekeeping gene GAPDH (used as a normalizing control) in independent wells. The data were analyzed using the comparative CT method (User Bulletin No. 2, Perkin Elmer Life Sciences) [67] and represented the relative mRNA levels for each transcript.

### Co-IP and peptide blocking experiments

Whole cerebellums from WT and *Fmr1* KO mice were homogenized in ice-cold Pull-Down buffer (1% Lubrol, 50 mM Tris–HCl, pH 7.4, 10 nM Na-orthovanadate, 30 mM Na-pyrophosphate, 50 mM NaF, 20 μm ZnCl_2_, and 0.25 mM PMSF, with the addition of protease inhibitor cocktail) using a Potter-Elvehjem grinding chamber (VWR). Samples were then centrifuged at 5000*g* for 20 min and 500 μL of supernatants were transferred into 1.5 ml Eppendorf tubes. 1:100 dilutions of anti-FMRP (Abcam 17722), anti-Kv1.2 (K14/16; Neuromab), anti-Kv1.2 ext-c (extracellular; Alomone labs), anti-Kv1.2 N-term (NBP1-42802; Novus), or anti-GAPDH (GAPDH-71.1; 1:80,000; Sigma-Aldrich) antibodies were added to the homogenate and incubated at 4 °C under continuous agitation for 2 h. A total 30 μL of a 1:1 mixture of pre-washed protein A and protein G magnabeads (Genscript) was then added and the samples were incubated under the same conditions for another 2 h. Beads were washed 4 times with PBS-Tween (0.2% Tween w/v) and then centrifuged at 3000*g* for 1 min. A sample was kept to verify total protein input. Protein was disassociated from the beads with the addition of 30 μL of 2× loading buffer (4% SDS, 20% glycerol w/v, 0.1 M dithiothreitol, 100 mM Tris–HCl, pH 6.8) then submitting the samples to 3 min at 95 °C. Samples were run on SDS-PAGE and transferred onto nitrocellulose membranes. Samples were loaded at equal volumes in each gel and probed for either target protein independently (FMRP or Kv1.2). Anti-Kv1.2 (K14/16; Neuromab) and anti-FMRP (5c2; BioLegend) were used to reveal Kv1.2 and FMRP, respectively. Bands were quantified as previously described using the AlphaEase SA software.

To map the interaction between Kv1.2 and FMRP, we designed a series of peptides with identical sequences of different segments of the C-terminus of Kv1.2 using solid-phase peptide synthesis by GenScript USA Inc. PKIP**p**S**p**SPDLKKSRSASTISKSD and PKIPSSPDLKKSRSASTISKSD corresponding to amino acids 436–457 of the mouse Kv1.2 protein sequence were obtained from NCBI (NCBI Reference Sequence: NP_032443.3). Scrambled peptide was generated from the bi-phosphorylated peptide sequence using the randomizer function (from www.random.org) to obtain IKPSTLSDSRAK**p**SSDP**p**SKPSIK. Peptides were prepared at high-purity (>95%; Canada Peptide). All peptide were maintained desiccated at −20 °C and freshly dissolved in Millipore H_2_O or in the appropriate experimental buffers as indicated. For the peptide PD competition, a concentration of 250 μM was added prior to the anti-FMRP antibody and incubation was carried out as indicated above. Each experiment was performed at least in triplicate.

### Behavioral tests and DHA treatment

As previously described [17], acoustic startle response was measured from 7–9-week-old mice, which were submitted to no pulse or a pulse stimulation (120 dB, 40 ms) alone or in combination of a prepulse (either 72, 78, or 82 dB, 20 ms). Maximal startle response was recorded and compared between groups for all types of trials. The sound attenuated chambers and analysis software were obtained from San Diego Instruments (San Diego, USA). One week prior to testing, mice were fed with normal chow or calorically equivalent chow enriched with 0.5% DHA (Sigma) prepared by Research Diets (New Brunswick, USA). Diets were prepared with identical dyes to be indistinguishable by eye. Mice were tested for acoustic startle response only once between 4 p.m. and 7 p.m.

The three-chambered sociability test was composed of three sessions: habituation (trail 1), sociability (trial 2), and social novelty (trial 3), which evaluated sociable behavior and recognition of social novelty [46]. For habituation, 7–9-week-old testing male mouse was placed in the middle chamber and allowed to freely explore all three chambers. In the sociability session, a stranger mouse (the same strain and gender but never encountered before) was put underneath a grid cup and placed in one of the two side-chambers, while another identical empty cup was placed on the other side. In the social novelty session, another stranger mouse was put inside the previously empty cup. Each session lasted 9 min. Physical contacts around the cups with the nose, head, and forelimbs, were defined as sniffing behavior and used as an index for sociable behavior [47]. A camera, connected to a computer, was used to track mice activity using the ANY-maze software (ANY-maze, Stoelting Co., IL, USA). Testing was conducted 30 min after saline or DHA (200 mg/kg, Sigma) injection (intraperitoneal, i.p.) to minimize distress from drug administration and ensure normal physical activity that could be affected by DHA for a short period.

### Statistics

Statistical tests of significance were two-tailed, paired or unpaired Student’s *t* tests, with a *p* value cut-off of <0.05. Data were expressed as the mean ± standard error (s.e.m.) from a population of cells, samples, or animals (*n*). Repeated two-way ANOVAs were conducted for the analysis of performance in the three-chambered test with the within-group factors “treatment” and “stranger”. Repeated one-way ANOVAs were applied in all other tests. Data analysis on variance and normal distribution were performed using GraphPad Prism 5 (GraphPad Software; La Jolla, CA, USA). Experimenters were blind to the treatment group in the behavioral testing and the groups were only revealed after the data was analyzed. Sample sizes were determined by previous studies using similar experimental protocols [17, 28].

### FMRP expression and purification

Small Ubiquitin-like Modifier (SUMO) fusion construct containing cDNA for full-length human FMRP was generated by gene synthesis (GenScript USA Inc.) and subcloned into a pET SUMO vector (Invitrogen). Expression of full-length FMRP from bacterial cells has been shown to prevent sample heterogeneity due to post-translational modifications such as phosphorylation and arginine methylation [68]. The pET SUMO–FMRP construct was transformed into *Escherichia coli* BL21 (DE3) Codon Plus cells and grown at 37 °C in Lennox broth medium. Protein expression was induced with 0.5 mM isopropyl-β-D-thiogalactopyranoside when optical density reached ~0.6, followed by 18 h of additional growth at 16 °C. Cells were harvested by centrifugation and pellets stored at −20 °C. In order to purify SUMO–FMRP, thawed pellets were resuspended in lysis buffer [50 mM Tris, pH 8.0, 500 mM NaCl, 20 mM imidazole, 10% glycerol, 250 mM arginine, 5 mM benzamidine, and 2 mM dithiothreitol (DTT)], supplemented with DNAse 1 and cOmplete^™^ Protease Inhibitor Cocktail (Sigma-Aldrich). After cell lysis and centrifugation, the filtered supernatant was loaded onto a 20 ml NiNTA column equilibrated in the same lysis buffer. The column was extensively washed with 20 column volumes (CV) of the same buffer supplemented with 100 mM imidazole. Elution of SUMO-fusion FMRP was performed using 100–500 mM imidazole gradient, and protein-containing fractions were combined and a 6× His tagged Ulp protease was added. Cleavage of the His-SUMO tag was performed overnight in a cold room (7 °C) while dialyzing in 4 L of 50 mM Tris, pH 8.0, 500 mM NaCl, 50 mM imidazole, 10% glycerol, 250 mM arginine, 5 mM benzamidine, and 2 mM DTT. Complete cleavage of the SUMO tag by Ulp was confirmed by SDS-PAGE and the protein of interest was separated from the Ulp protease and SUMO tag by passing the cleavage reaction over a second NiNTA column. Flow-through and wash fractions containing protein of interest were combined and then loaded onto a Superdex 200 gel filtration column pre-equilibrated with the same lysis buffer.

The eluted purified proteins were analyzed by both SDS-PAGE and mass spectrometry, and the combined fractions were dialyzed three times in 5 L of Fluorescence Binding Buffer (100 mM NaCl, 50 mM Na_2_PO_4_ pH 7.4, 2 mM DTT). After filtering through a 0.22 μm GV Durapore Centrifugal Filter Unit (Millipore), FMRP concentration was determined by absorbance at 280 nm using a molar extinction coefficient of 48,360 M^−1^ cm^−1^, and the protein was used for binding studies with Kv1.2 C-terminal peptides.

### In vitro fluorescence binding assay

All measurements were carried out in the Fluorescence Binding Buffer at 20 °C using a PTI Quanta Master 80 Spectrofluorometer (HORIBA Scientific) by monitoring intrinsic protein fluorescence with an excitation wavelength of 287 nm. Prior to performing a binding titration for any of the peptides, a full emission spectrum (300–450 nm) of the peptide:FMRP complex was compared to that of apo-FMRP. Differences in the spectrum of the complex (500–1000 μM peptide and 1 μM FMRP) and apo-FMRP at the same concentration (i.e., fluorescence quenching, enhancement or shifts of the emission maximum) indicated binding. For each of these peptides, binding measurements were performed by adding aliquots of saturated 1 µM FMRP:peptide complex (titration solution) to 400 μL of 1 μM apo-FMRP solution as described by Wells and Di Cera [69]. Briefly, at each titration step, a volume *v*_*D*_ was removed from the cuvette containing the apo-FMRP and replaced by an equal volume of the titration solution (1 μM FMRP: 200–500 μM peptide complex). As a result, the concentration of FMRP (1 μM) and total volume of solution in the cuvette, *V* = 400 μL, remained constant while the concentration of the peptide at the *i*th titration step, [peptide]_*i*_, was increased, and given by

[peptide]_*i*_ = [peptide]_0_^*i*^.*D*^*i*^+[peptide]_∞_^*i*^.(1 − *D*^*i*^),

where, [peptide]_0_ was the initial peptide concentration in the cuvette, [peptide]_∞_ was the peptide concentration in the titration solution, and *D* was the dilution factor, *D* = (*V* − *v*_*D*_)/*V*.

For each titration point, emission scan between 325 and 345 nm, in increments of 1 nm, was collected in triplicates, from which the average was obtained at each wavelength. These average fluorescence intensity values were then normalized between 1 (apo-FMRP) and 0 (saturated peptide-bound FMRP), and the average and standard deviation from all the wavelengths were then calculated.

### Model simulations for FMRP–Kv1.2 C-motif docking

Prior to docking, geometric (groove/ridges) and/or physico-chemical (electrostatic/hydrophobic) complementarities between FMRP and the Kv1.2 peptides that could potentially be a binding interface were identified using the PyMOL Molecular Graphics System (Version 1.7.4 Schrödinger, LLC) and the Poisson–Boltzmann solver from the Im lab.

To perform this electrostatic-based docking, an extended structure of the doubly-phosphorylated 22-residue Kv1.2 peptide was first generated using a homemade Crystallography and NMR system (CNS) [70] script based on ARIA2 [71]. Then a rigid body docking was performed using CNS with ambiguous restraints on the position of the PpSpSP motif into the basic groove of the FMRP Age1–Age2–KH0 domain (PDB 4QW2) [39] as a monomer, while the remainder of the Kv1.2 peptide was left unrestrained. In addition, we tested 180° rotation for each docked peptide about the phosphate positions. Out of a total of 5000 runs, the lowest energy complex was then selected for subsequent manual wrapping of Kv1.2 peptide around FMRP using PyMol [72] and its embedded CHARMM force field [73]. Again, while the positions of the two phosphoserines were kept fixed, the peptide was guided around the FMRP surface using the Sculpting option of PyMol that utilized all energy terms. Three different pathways of the peptide along the surface of FMRP were generated, which were used as starting points in the docking program HADDOCK [74] for minimizing and optimizing the position of Kv1.2 peptide at the surface of FMRP, with 10 simulations done per initial complex. For the docking protocol, the peptide was set as “fully flexible” and the flexible part of FMRP was set as “automatic”, such that only the part of FMRP located in the vicinity of the peptide was flexible. The complex with the best HADDOCK score was selected, revealing three potential modes of engagement between the doubly phosphorylate Kv1.2 peptide and FMRP. With such manual and automatic sequential fitting strategies, we obtained three different models in which lowest energy complexes were achieved, depending on the energy terms that were taken into account (Supplementary Figure 7). Following this docking, we generated a dimer model for each by aligning Cα atoms of the Age1–Age2–KH0 domain-containing unit onto each of two chains within the dimeric 4OVA structure (3.0 Å resolution structure of Age1–Age2–KH0 of FMRP). Structures were deposited online.

## Electronic supplementary material

Supplementary Figures & Table (clean)

2. Lowest Haddock Dimer

3. Lowest Overall Dimer

1. Lowest Elec Dimer
