# Supplementary material for: Identification of a molecular locus for normalizing dysregulated GABA release from interneurons in the Fragile X brain
Source: Mol Psychiatry. 2018 Sep 17;25(9):2017–35. doi: 10.1038/s41380-018-0240-0 (PMC7473840; doi:10.1038/s41380-018-0240-0)
Supplement: Supplementary file 1 — Supplementary Figures & Table (clean) [file 41380_2018_240_MOESM1_ESM.pdf]

Supplementary Information

Supplementary Figure 1

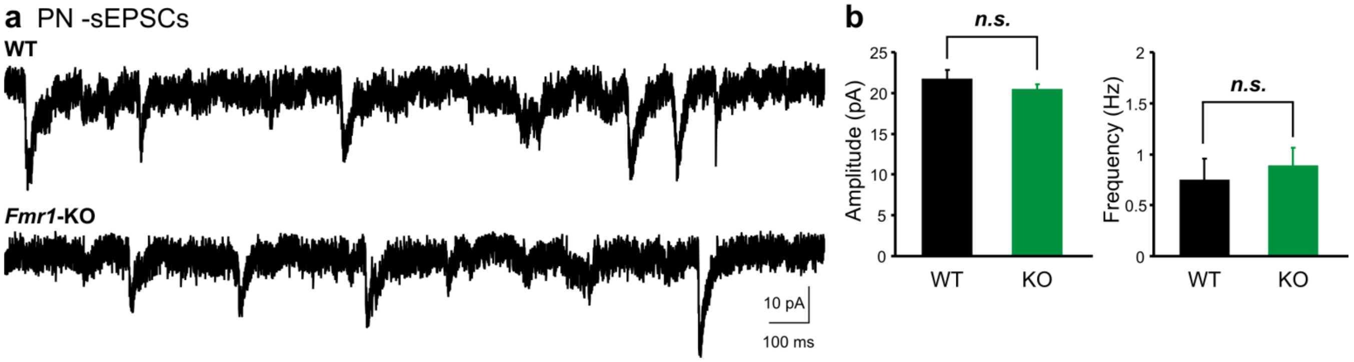

Supplementary Figure 1. (**a**) Representative recordings of sEPSCs from PNs by blocking inhibitory inputs with bicuculline (10 μM) in WT (black) and *Fmr1*-KO (green) brain slices. (**b**) Summary of the amplitude (left) and frequency (right) of sEPSCs for WT (black bar, n=6) and KO (green bars, n=5) groups.

Supplementary Figure 2

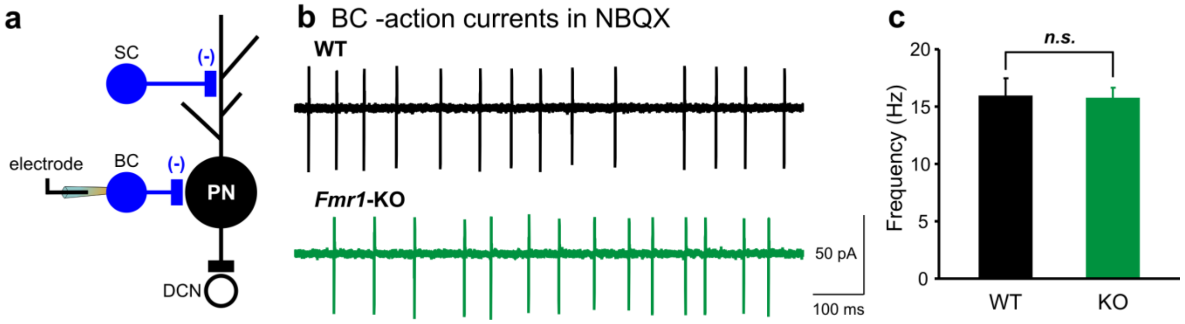

Supplementary Figure 2. (**a**) Schematics to show the recording configuration from a basket cell (BC) in NBQX to isolate inhibitory input. (**b**) Cell-attached patch-clamp recordings of APs from a WT (black) and KO (green) BC soma. (**k**) Summary of BC firing frequency for WT (n=11) and KO (n=9) genotypes.

Supplementary Figure 3

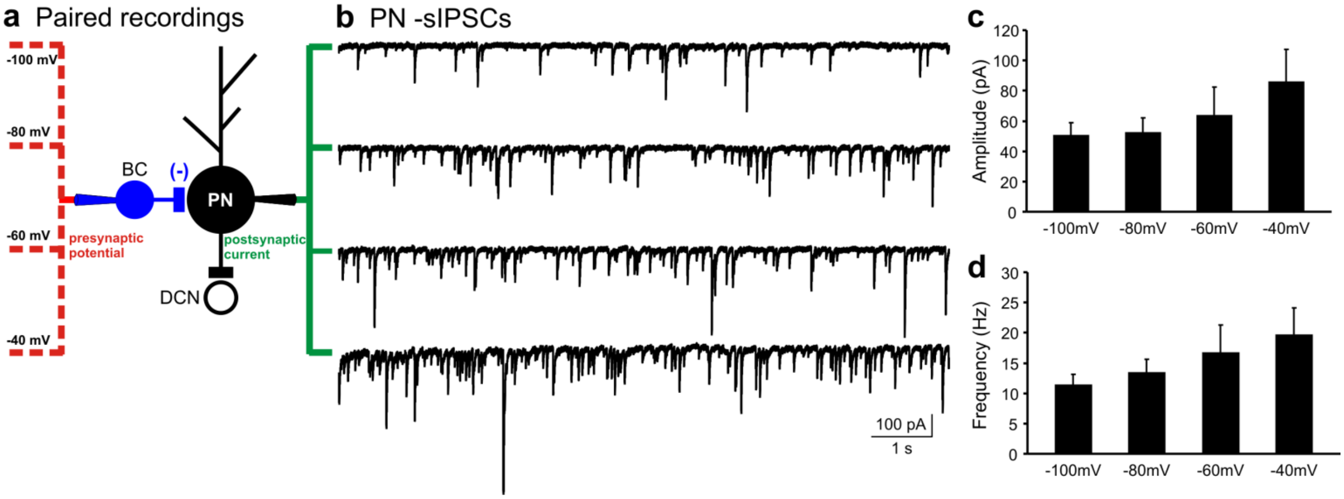

Supplementary Figure 3. (**a**) Schematics of simultaneous paired recordings from BCs and PNs. (**b**) sIPSCs recorded from PNs responding to varied holding potentials of BCs (-40 to -100 mV at 20 mV increment). Depolarization of the soma in the voltage-clamp mode was strategized to promote the escape of distal axonal terminals from space-clamp and action potential firings to enhance GABA release. The larger and more frequent sIPSCs reflected AP and Ca^{2+}-dependent synchronous and asynchronous release, both of which superimpose onto mIPSCs. (**c,d**) Summary of amplitude and frequency of sIPSCs elicited by holding BCs at different potentials (n=6).

Supplementary Figure 4

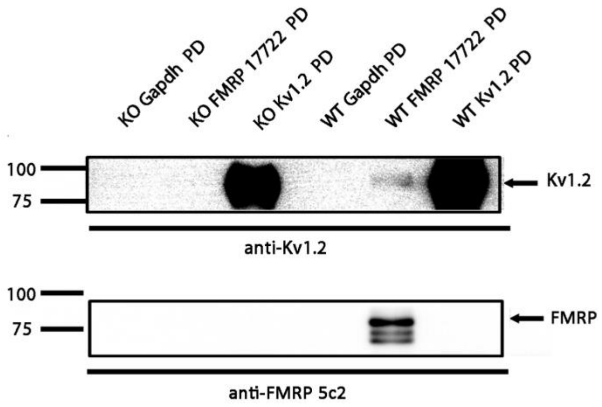

Supplementary Figure 4. Protein immunoprecipitation of the whole cerebellum by anti-GAPDH, anti-FMRP (5c2) and anti-Kv1.2 (K14/16) from WT and *Fmr1*-KO mice as seen on western immunoblot. The input and immunoprecipitated fractions (IP) of Western blots were from whole cerebellums.

Supplementary Figure 5

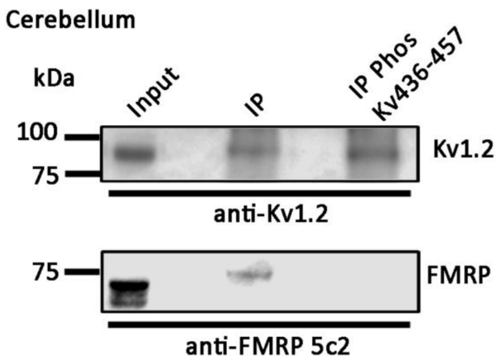

Supplementary Figure 5. Co-IP of Kv1.2 (top) and FMRP (bottom) using Kv1.2 antibody recognizing its extracellular domain (anti-Kv1.2 ext-c, Alomone labs) in the absence or presence of the phosphorylated Kv1.2 peptide (PhosKv436-457). The input and immunoprecipitated fractions (IP) of Western blots were from whole cerebellum of WT mice.

Supplementary Figure 6a

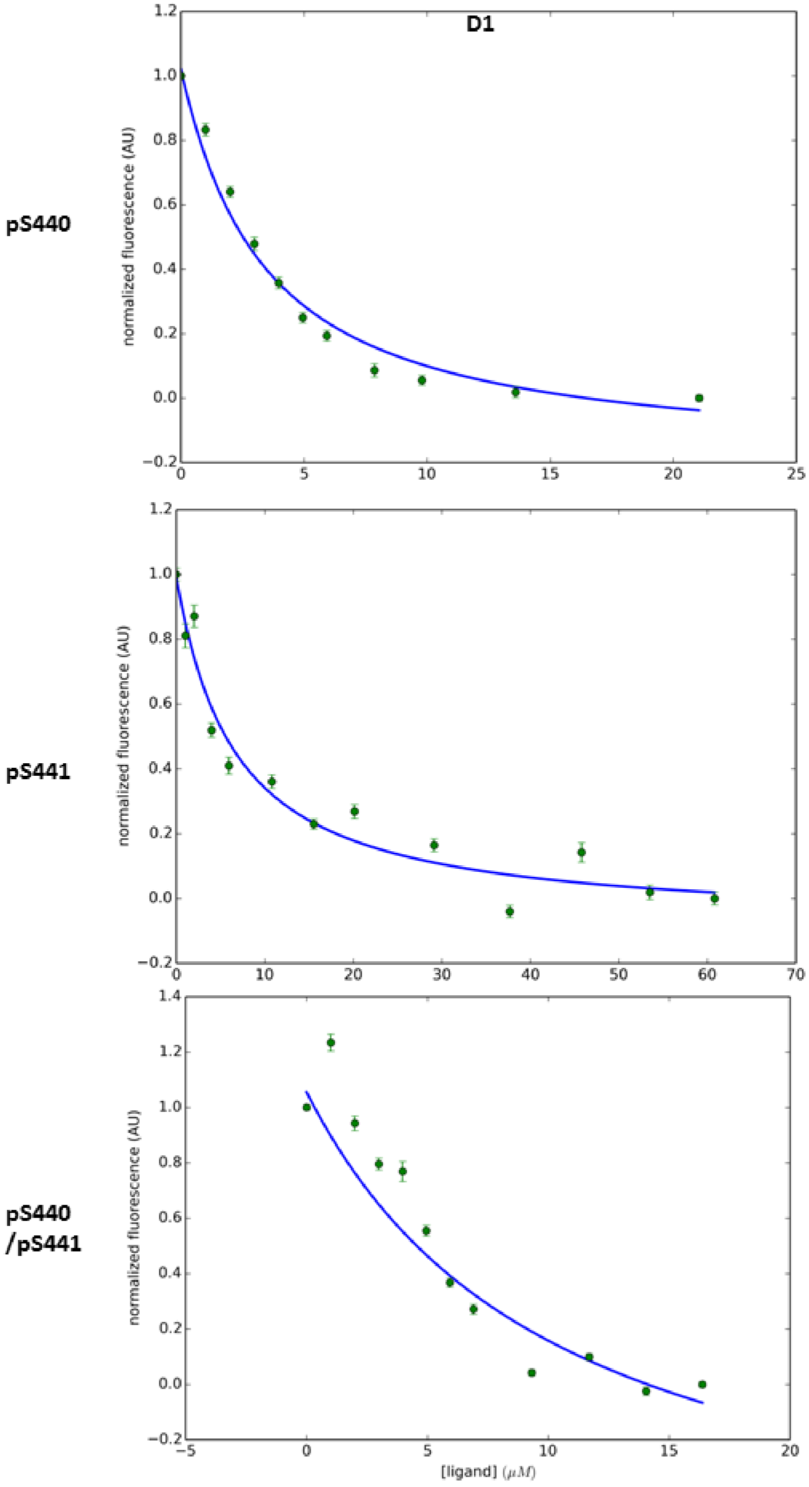

pS440

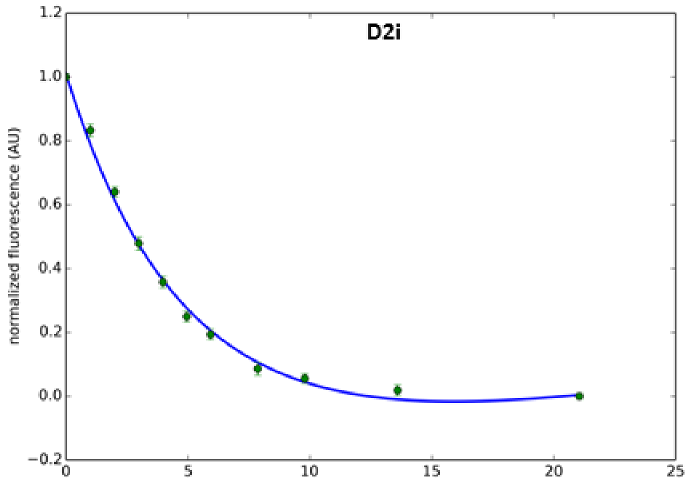

pS441

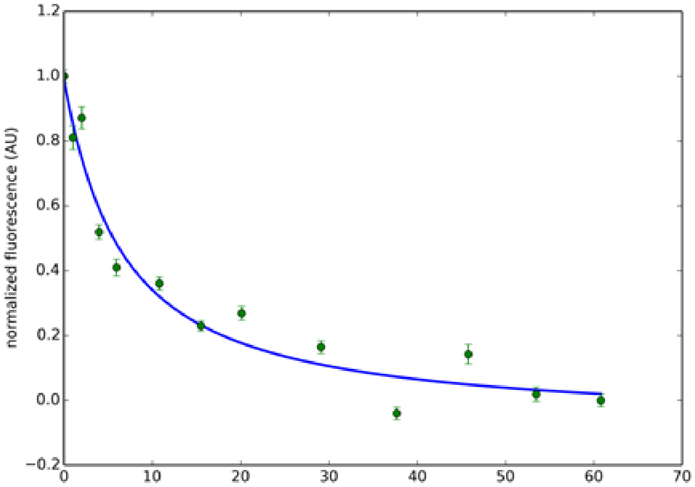

pS440 /pS441

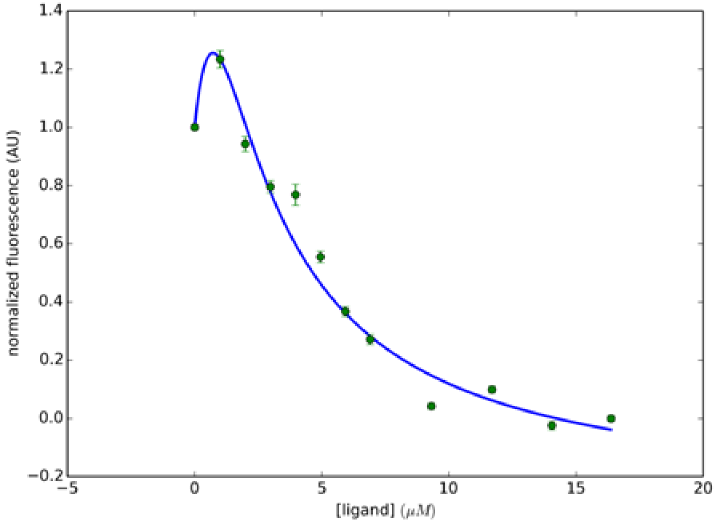

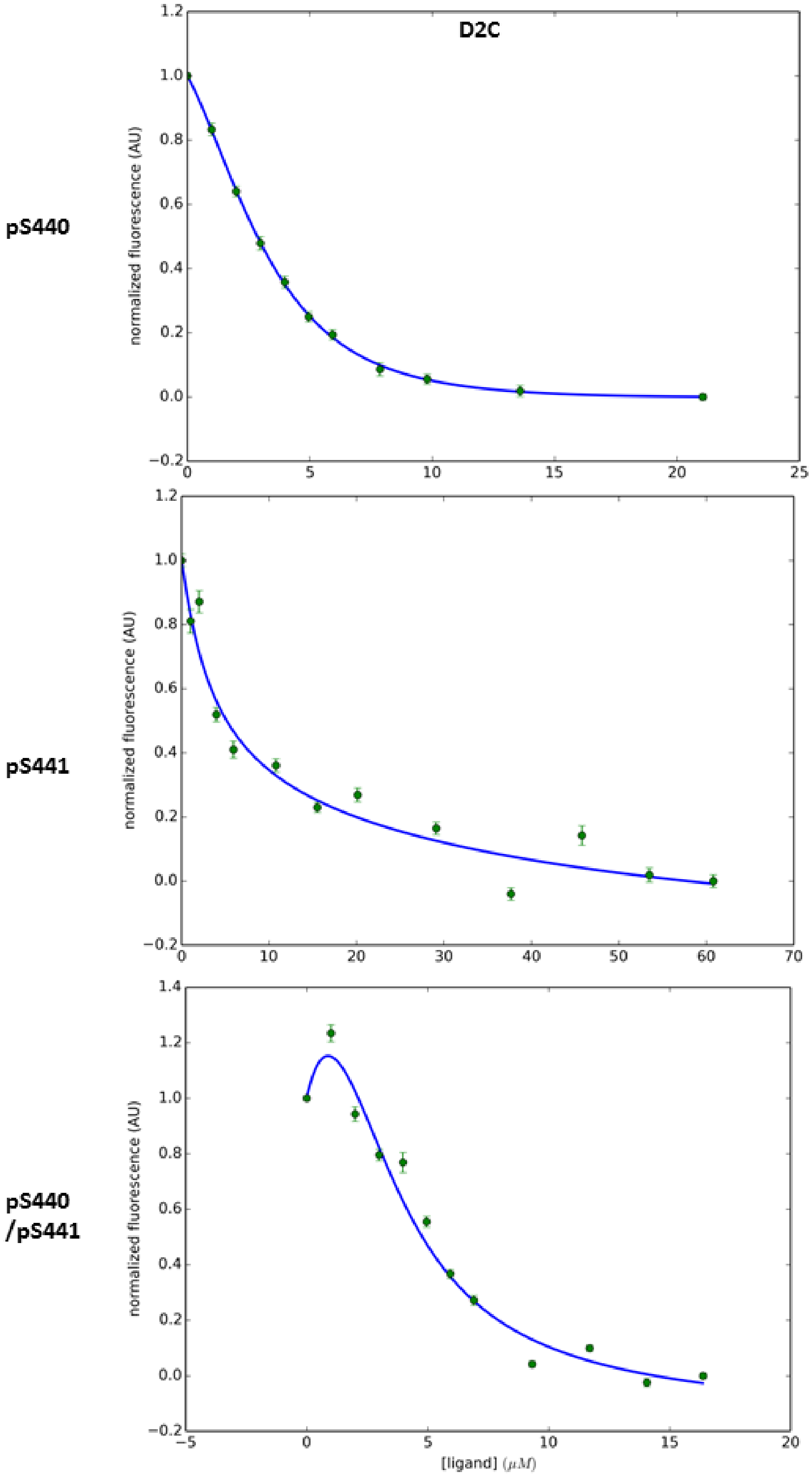
D2C
pS440
normalized fluorescence (AU)
pS441
normalized fluorescence (AU)
pS440
/pS441
normalized fluorescence (AU)
[ligand] (μM)

Supplementary Figure 6b

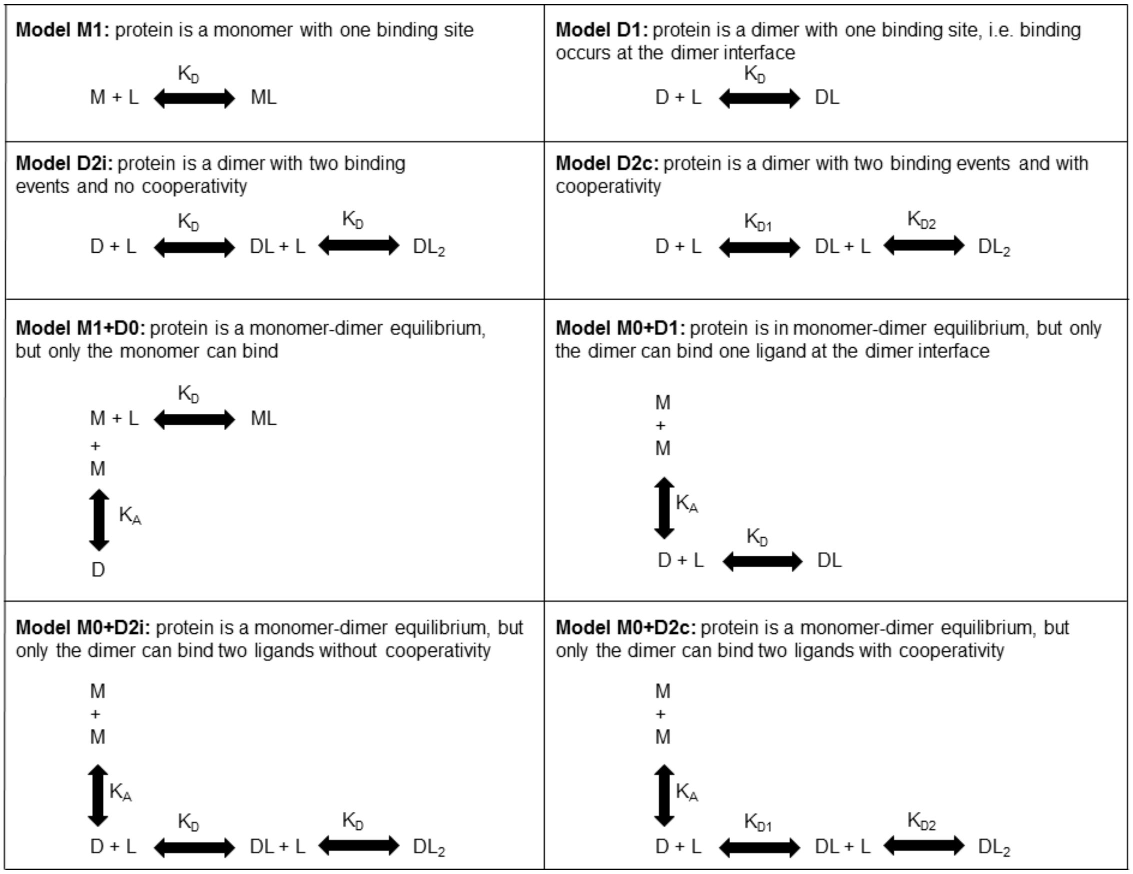

Supplementary Figure 6 (**a**) Binding profiles of pS440, pS441 and pS440/pS441 peptides; (**b**) Fitting binding data with different monomer or dimer models, D1, D2i and D2c.

In model **M1**, FMRP exist as a monomer with a single binding site, while in model **D1**, it exists exclusively as a dimer with only one binding site (for instance, binding occurs at the interface). In model **D2i,** FMRP is a dimer with two independent binding sites, one on each protomer (i.e. no co-operativity), but in model **D2c**, there is co-operativity between the two protomer binding sites. Therefore, the two dissociation constants (K_D) are identical in model **D2i**, while in **D2c** they are different (K_{D1} and K_{D2}) since the binding at the first site affects the binding at the second. Finally, we tested models in which apo-FMRP exists in monomer: dimer equilibrium but only the monomer or the dimer binds a single peptide ligand in models **M1+D0** and **M0+D1**, respectively, or in the models **M0+D2i** and **M0+D2c**, where only the dimer binds two peptide ligands, in an independent and co-operative mechanism, respectively. In order to distinguish which model best fits the binding data for each peptide, χ^2 statistics were compared[5] and fits which give physically unrealistic values (e.g. such as negative K_D values) were discarded.

According to the binding data analysis, the models in which full-length FMRP exists exclusively as a dimer give the best fits (Figure 4 and Table 1). The goodness of fit, indicated by the χ^2 / reduced χ^2 values, for the pS440 peptide binding data progressively improves from 92.8/11.6 to 16.4/2.3 to 0.99/0.16 for the **D1**, **D2i** and **D2c** models, respectively. This implies that pS440 binds dimeric FMRP in a co-operative mechanism (**D2c** model) with dissociation constants K_{D1} and K_{D2} of 33.9 ± 6.2 μM and 0.42 ± 0.08 μM, respectively, indicating that the binding affinity of the second ligand has been enhanced ~80-fold by the binding of the first ligand. The other models tested showed worse goodness of fit (data not shown). Interestingly, however, each of the other two phospho-peptides (pS441 and pS440/pS441) did not produce similar co-operative binding effects, suggesting that these peptides bind by a different mechanism. For example, the pS441 data could not be fitted to the **D2c** model with physically realistic values (i.e., fit produce a negative K_{D1} value) and were best fitted by models **D1** and **D2i**, which cannot be clearly distinguished based on their respective χ^2 and reduced χ^2 (Supplementary Table 1). Furthermore, the two models produced similar K_D values of 6.34 ± 1.48 μM and 6.52 ± 0.02 μM, respectively. Interestingly, these values are ~5-fold tighter than the K_{D1} of the pS440 and ~15-fold weaker than the K_{D2}. Finally, the pS440/pS441 peptide showed similar lack of co-operativity but with tighter binding affinities for each protomer (K_D = 1.08 ± 0.26 μM).

Supplementary Table 1. Parameters and χ^2 statistics for fitting the different phospho-peptides to FMRP

**pS440**	**D1**	**D2i**	**D2c**
K_{D1} (μM)	3.09 ± 0.46	15.7 ± 2.6	33.9 ± 6.2
K_{D2} (μM)	-	15.7 ± 2.6	0.42 ± 0.08
K_A (μM)	-	-	-
χ^2 / reduced χ^2	92.8/11.6	16.4/2.3	0.99/0.16
**pS441**	**D1**	**D2i**	**D2c**
K_{D1} (μM)	6.34 ± 1.48	6.52 ± 0.01	N/A
K_{D2} (μM)	-	6.52 ± 0.01	N/A
K_A (μM)	-	-	N/A
χ^2 / reduced χ^2	106/10	107/11	N/A

**pS440/pS441**	**D1**	**D2i**	**D2c**
K_{D1} (μM)	10.1 ± 4.4	1.08 ± 0.26	N/A
K_{D2} (μM)	-	1.08 ± 0.26	N/A
K_A (μM)	-	-	N/A
χ^2 / reduced χ^2	536/59.6	166/20.7	N/A

Supplementary Figure 7

Model 1: HADDOCK Score and Van der Waals Energy

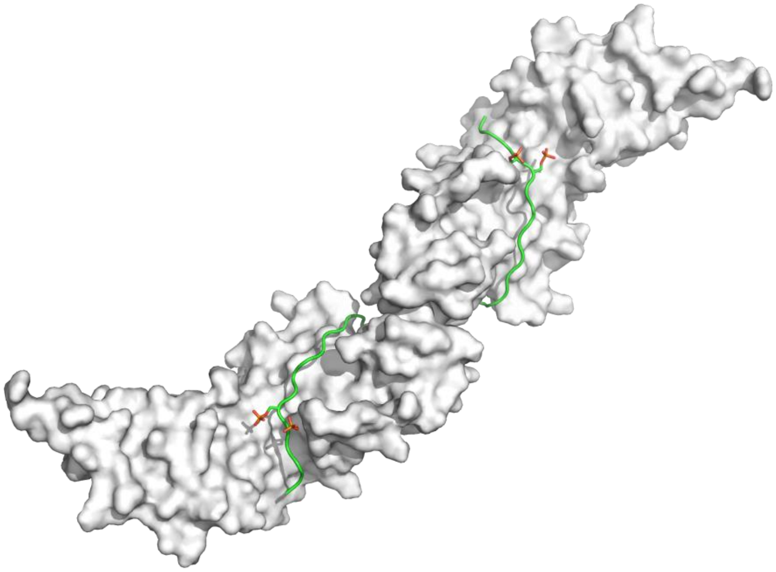

Model 2: Lowest Electrostatic and Van der Waals Energy

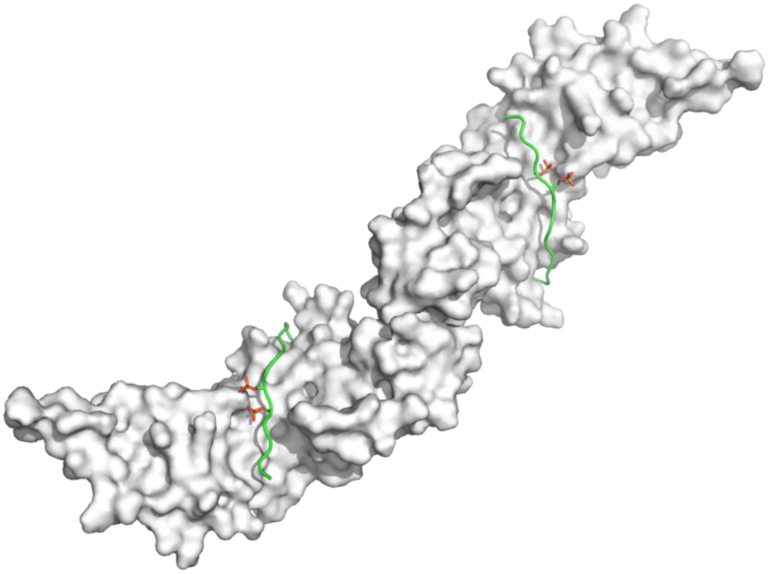

Model 3: Lowest Overall Energy

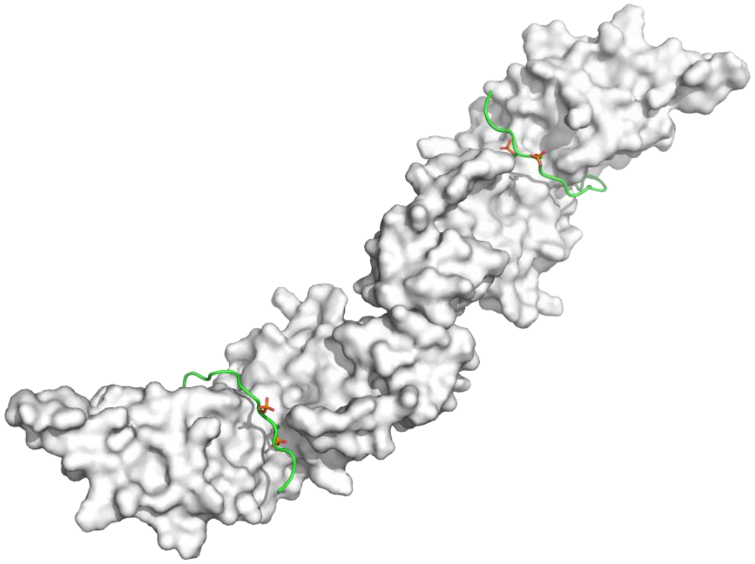

Supplementary Figure 7. Different Kv1.2 Phospho-peptide:FMRP Binding Models. **Model 1** has the lowest HADDOCK score and also the lowest Van der Waals energy term; **Model 2** has the lowest electrostatic energy conformer, and also the lowest Van der Waals plus electrostatic energy term, with the peptide being fit onto FMRP so that the phospho-serines are not located as in the starting complex; **Model 3** has the lowest overall energy complex with all bonds, angles, dihedrals, Van der Waals, electrostatic energy being taken into account.

Supplementary Figure 8

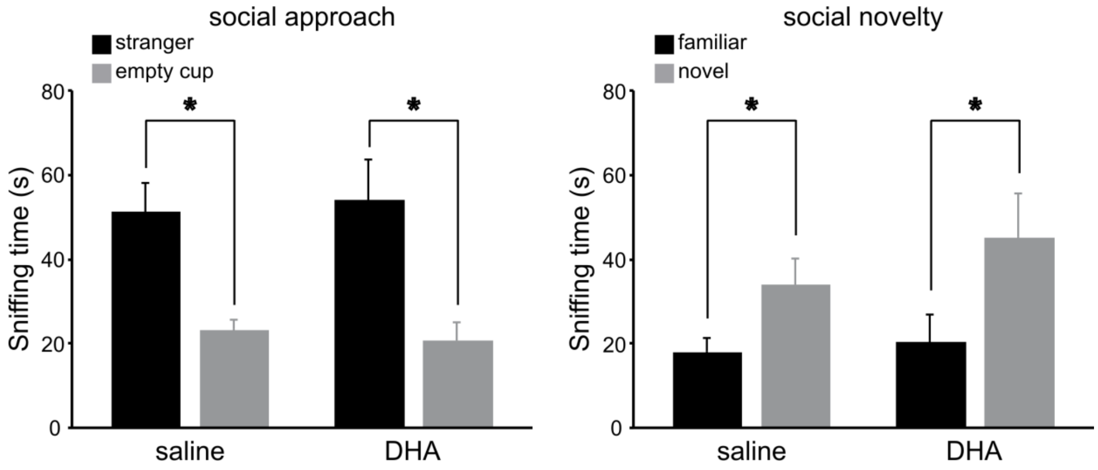

Supplementary Figure 8. DHA treatment did not affect social performance of WT mice in the three-chambered test. Sniffing time spent on the stranger and empty cup in the sociability session (left); and spent on the novel and familiar mouse in the social novelty session (right) during the tests were

summarized for male C57BL/6J mice (n=8) injected with saline or DHA (200 mg/kg). Repeated two-way ANOVAs were applied with the factors “drug” and “object” to analyze the data. In social approach trial, there was a significant “object” effect ($F_{1, 7} = 81.675$, $p<0.001$), but not “drug” or their interaction effect ($p>0.05$). Both groups spent more time sniffing the stranger than the empty cup (saline: $t_7=4.499$, $p=0.003$; DHA: $t_7=5.123$, $p=0.001$, by paired t-tests). In social novelty trail, a significant effect of “object” ($F_{1, 7} = 16.088$, $p=0.005$), but not “drug” or their interaction ($p>0.05$), was found. Both groups spent more time sniffing the novel stranger than the familiar one (saline: $t_7=3.945$, $p=0.006$; DHA: $t_7=3.223$, $p=0.015$, by paired t-tests). No significant differences were found in the analysis of distance travelled during the trials by repeated one-way ANOVAs ($p>0.05$).
